# Further reduction in soil bacterial diversity under severe acidification in European temperate forests

**DOI:** 10.1111/ejss.70005

**Published:** 2024-11-08

**Authors:** Mélody Rousseau, Andjin Siegenthaler, Andrew K. Skidmore, G. Arjen de Groot, Ivo Laros

**Affiliations:** ^1^ Natural Resources Department, Faculty of Geo‐Information Science and Earth Observation University of Twente Enschede The Netherlands; ^2^ Wageningen Environmental Research Wageningen University & Research Wageningen The Netherlands

**Keywords:** bacterial diversity, bacterial putative function, forest health, forest soil, nitrogen deposition, pH

## Abstract

Despite a decrease in industrial nitrogen and sulfur deposition over recent decades, soil acidification remains a persistent challenge to European forest health, especially in regions of intense agriculture and urbanisation. Using topsoil eDNA metabarcoding and functional annotations from a sample of 49 plots (each 30 × 30 m) located in The Netherlands and Germany, we investigated the effect of severe acidification on bacterial taxonomic diversity under different forest types and explored potential functional implications for nutrient cycling. Furthermore, we assessed which soil parameters known to influence soil bacterial communities affect these acidophilic communities. Here, we are the first to demonstrate under natural conditions that soil bacterial diversity in extremely acidic soils (pH <4.5) continues to decline similarly across forest types as pH further decreases under intensifying human activity. Our results confirmed pH as the key driver of soil bacterial communities, even in extremely acidic soils. Ongoing severe acidification continues to reduce bacterial communities, favouring taxa adapted to extreme acidity and primarily involved in recalcitrant carbon‐degradation compounds (e.g. cellulolysis potential = 0.78%–9.99%) while simultaneously diminishing taxa associated with nitrogen cycling (e.g. fixation potential = 6.72%–0.00%). Altogether, our findings indicate a further decline in bacterial diversity in already extremely acidic soils, likely disrupting nutrient cycling through changes in immobilisation and mineralisation processes. Our study highlights the continuous acidification of European temperate forests to extremely low pH levels, further disrupting forest ecosystem functioning. The significant reduction in bacterial diversity under such a severe acidification gradient, as demonstrated here, underscores the necessity to include severely acidified forests in conservation programmes and monitoring to prevent further degradation of European soils beyond repair.

**Highlights**

- We studied soil bacterial diversity changes in extremely acidic soils (pH <4.5).
- Soil bacterial diversity continues to decline along a gradient of severe acidification.
- Other soil properties, in addition to pH, limit bacterial diversity in extremely acidic soils.
- Our study highlights the necessity to include severely acidified forests in conservation programmes.

## 1 INTRODUCTION

Soil acidification in temperate European forests results from the deposition of human‐generated reactive nitrogen and sulfur compounds, which peaked in the 1980s (Lajtha & Jones, 2013). These compounds, including sulfur dioxide (SO_2_) and nitrogen dioxide (NO_2_), were released into the atmosphere due to fossil fuel combustion, including traffic emissions and energy generation, building construction and industrial processes (Lajtha & Jones, 2013). Additionally, agricultural intensification associated with the ‘green revolution’ (Pingali, 2012) has accelerated soil acidification through the excessive use of fertiliser (e.g. NH_4_NO_3_) and the production of manure from intensive animal husbandry (Barak et al., 1997; Skidmore et al., 2024). While European NO_X_ and SO_X_ emissions have declined substantially over the last decades (Fagerli et al., 2017; Vestreng et al., 2007), NH_3_ emissions stabilised after an initial decline in the 1990s due to ongoing agricultural intensification (EEA, 2020; Simpson et al., 2024; Skidmore et al., 2024). This raises concerns about the potential further elevation of acidity levels in some European temperate forests. This legacy of soil acidification, combined with ongoing and even intensifying acidification, poses a risk to the stability and resilience of forest ecosystems in Europe, particularly for temperate forests located in areas of intense traffic, agriculture and/or urbanisation (Dentener et al., 2006; Posch et al., 2015). This especially concerns sensitive forested areas, such as Natura 2000 areas that lack regular field monitoring (Abdullah et al., 2024).

Acidification of forest soils is associated with a reduction in soil biodiversity (Hu et al., 2024). For microbial communities, increased acidification shifts the community composition towards a simpler (i.e. reduced overall diversity) oligotrophic (i.e. bacteria found in environments with low carbon accessibility) acid‐tolerant community (Choma et al., 2020; Ramirez et al., 2012). These changes in the metabolic needs and capacities of the selected microorganisms result in notable alterations in enzymatic activities and gene expression involved in nutrient cycling (Lladó et al., 2018). For instance, Hagh‐Doust et al. (2023) demonstrated less diverse nitrogen and carbon pool genes as the soil acidifies, partially linking this to lower microbial diversity. Past research suggests a tipping point at a pH of approximately 4.5, resulting in a highly specialised and dissimilar community compared to soils with higher pH (Rothenberg et al., 2022; Rousk et al., 2009). For example, by inducing aluminium toxicity and altering nutrient availability, an extremely acidic soil habitat (pH <4.5; Batjes, 1995) acts as an environmental filter where only a few microbial taxa can thrive (Rousk et al., 2009).

The nature of stands (e.g. deciduous vs. coniferous) has a key role in determining forest soil pH (Handley, 1954). Leaf litter from deciduous trees tends to decompose relatively faster, enhancing base saturation and increasing the pH of the topsoil (Bani et al., 2018). In contrast, litter decomposition of coniferous trees often leads to soil acidification, primarily due to the higher release of organic acids (e.g. polyphenols) from their needles (Ahrends et al., 2022; Hornung, 1985). Furthermore, coniferous trees capture more atmospheric elements, such as nitrogen and sulfur, thereby increasing the acidification rate (Augusto et al., 2002). By modifying soil pH but also other physical–chemical parameters such as organic matter, carbon, nitrogen and phosphorus contents, trees actively shape and alter the soil microbiome (Buresova et al., 2019; Kopáček et al., 2013). For example, Bárta et al. (2017) showed that bacterial communities in coniferous and deciduous stands recovering from past acidification differed and exhibited distinct denitrification potential.

So far, studies investigating pH effects on soil microbiota have often focused on broad soil pH gradients over extensive areas (e.g. across biome; Kaiser et al., 2016; Lauber et al., 2009; Nacke et al., 2011) or at very local scale field experiments based on the addition of either neutralising experiments (liming) or acidifying agents (e.g. Choma et al., 2020; Tahovská et al., 2020). However, it remains uncertain whether such trends in soil bacterial diversity continue under even more severe acidification in natural conditions, that is among extremely acidic soils with pH <4.5 (Batjes, 1995). Exploration of changes in this pH spectrum is especially relevant as soil pH values below 4.5 are currently being reported for European temperate forests (Abdullah et al., 2024; Šantrůčková et al., 2019; Skidmore et al., 2024). This is especially relevant for bacterial communities as they appear more sensitive to soil acidification than fungi (Chen et al., 2022; Choma et al., 2020). For fungi, the effects of pH are indirect, such as changes in nutrient availability and increased competition with bacteria (Rousk et al., 2010). In contrast, soil pH directly influences bacteria by causing physiological stress (Rousk et al., 2009).

The overall aim of this study is to investigate the response of topsoil bacterial communities across different forest types in two European mixed‐temperate forests—one in The Netherlands and the other in Germany—to a gradient of severe acidification (pH <4.5), as reported for European temperate forests (Abdullah et al., 2024; Šantrůčková et al., 2019; Skidmore et al., 2024). Additionally, we aimed to identify which common soil biochemical drivers influence these acidophilic communities in the same European mixed‐temperate forests. Using metabarcoding, we explored the effect of severe acidification on bacterial taxonomic diversity. Additionally, we examined potential implications for bacterial functional diversity through functional annotations of the same metabarcoding dataset. We hypothesised that:

- Severe acidification has a limited effect on soil bacterial taxonomical and functional potential diversity because acidophilic taxa have already been selected in extremely acidic soils.
- Bacterial communities in coniferous stands are less sensitive to severe acidification than those in deciduous stands.
- Other biochemical parameters may further limit and shape bacterial communities as bacterial communities have already been selected under acidic conditions.

## 2 MATERIALS AND METHODS

### 2.1 Study site description

**FIGURE 1:**
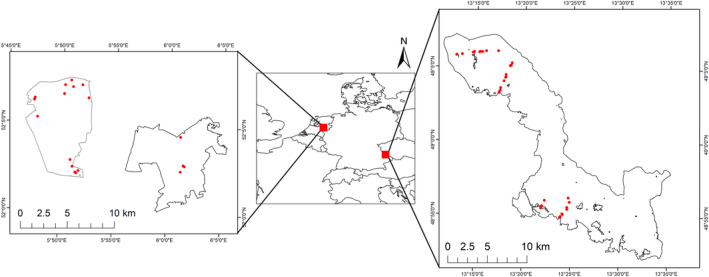
Sampled regions and plot locations in Germany (Bavarian NP) and The Netherlands (Veluwe NP).

Soil samples were collected during the summer of 2020 (May–August) in two forested regional landscapes (Figure 1). The Bavarian Forest National Park is located in south‐east Germany at the border with the Czech Republic (48.9597° N, 13.3949° E, hereafter referred to as ‘Bavarian NP’). The park covers a total area of 24,000 ha and is influenced by continental and temperate climates (Heurich et al., 2010). With an elevational of 600 and 1450 m, the Bavarian NP is dominated by European beech (*Fagus sylvatica*) at lower altitudes and Norway spruce (*Picea abies*) at higher altitudes (Bässler et al., 2015). The parent material is gneiss and granite, developing into oligotrophic and acidic soils (van der Knaap et al., 2019). Specifically, the predominant soil types in the park are spodo‐dystric cambisol and dystric histosol (EC, 2004). The Bavarian NP is a system recovering from severe sulfur deposition in the 1980s, which resulted in the acute acidification of terrestrial ecosystems across large areas of central and eastern Europe (Baker et al., 2021). The park is currently subjected to a nitrogen deposition rate lower than 10 kg/ha/year and sulfur emissions lower than 1 mg/year (EEA, 2022; EMEP, 2024).

The Hoge Veluwe National Park (52.0787° N, 5.8325° E) and the Veluwezoom National Park (52.0483° N, 6.0199° E) are two national parks located in the central part of The Netherlands (hereafter referred to as ‘Veluwe NP’). The Veluwe NP is a mix of forest, heathland, drift sand and grassland ecosystems, covered by 14,000 ha of forest (Hein, 2011). It is characterised by a temperate maritime climate and an elevation of around 40 metres with little topographic variation. The Veluwe NP is dominated by a mix of Scots pine (*Pinus sylvestris*), Norway spruce (*P. abies*), English oak (*Quercus robur*), silver birch (*Betula pendula*) and European beech (*F. sylvatica*) growing on oligotrophic, sandy and acidic soils with low buffering capacity. The prevailing soil types include gleyic podzol, ferro‐humic podzol, orthic podzol and dystric regosol (EC, 2004). The parks are subjected to aerial deposition of sulfur dioxide (SO_2_) (<1 mg/year; EMEP, 2024) and nitrogen dioxide (NO_2_) from air pollution as well as high input of atmospheric ammonia (NH_3_) from surrounding intensive animal husbandry (de Hoop et al., 2022; Mol et al., 2003). The average nitrogen deposition rate is high, averaging 500 kg/ha/year (EEA, 2022).

### 2.2 Soil sampling design and collection

A total of 31 and 18 plots were collected in the forested areas of the Bavarian NP and the Veluwe NP, respectively. We used a stratified by vegetation cover strategy (Goedickemeier et al., 1997), modified by purposive sampling where access was poor and terrain difficult, to determine plot locations over forest type dominance (>75% of coniferous or deciduous tree dominance). Within each 30 m × 30 m plot, three 3 m × 3 m subplots were randomly selected, and one composite sample per subplot was collected, resulting in 93 samples (45 coniferous and 48 deciduous) for the Bavarian NP and 54 samples (30 coniferous and 24 deciduous) for the Veluwe NP. Each composite sample comprised nine soil cores (Ø 5 cm × h 10 cm, topsoil) collected following a systematic sampling design within a 3 × 3 m grid, minimising the effect of local heterogeneity (Taberlet et al., 2012). Alterations in the soil microbiome and soil conditions accompanying soil acidification can occur throughout a soil profile (Forstner et al., 2019). However, we focused exclusively on topsoil bacterial communities because this layer shows the strongest link between soil biodiversity, fertility and plant productivity (Delgado‐Baquerizo et al., 2017). To reduce the impact of litter‐specific microbial communities (Prescott & Grayston, 2013; Urbanová et al., 2015), we excluded the litter horizon prior to soil sampling. Consequently, all soil samples were taken from the organic and mineral horizons. To avoid DNA contamination between samples, soil sampling equipment was bleach‐sterilised and rinsed with demineralised water. Field controls (demineralised water used for rinsing the sterilised equipment) were collected every 15 samples. Each composite sample was manually homogenised, and large rocks and roots were removed. All samples were kept cool in the field and frozen to −30°C at the end of each sampling day until further analysis (<4 months).

### 2.3 Soil biochemical analysis

Samples were oven‐dried prior to biochemical measurements and ground with a ball mill (Planetary Ball Mill PM 200, Retsch). Soil pH (H_2_O) was measured with a pH meter (Metrohm 914 pH and conductivity meter) following the ISRIC protocol (Black, 1965; van Reeuwijk, 2002). Total nitrogen (TN) and total carbon (TC) were measured with a CHN analyser (Perkin Elmer 2400 CHN/O Series II System). C:N ratio (mass basis) was defined as the ratio between TC (%) and TN (%) following the ISRIC calculations (van Reeuwijk, 2002). Soil organic matter (SOM) was determined using the loss‐on‐ignition method (0.5 g of soil for 16 h at 550°C). Soil organic carbon (SOC) content was estimated with the following equation:

$$\mathrm{SOC}\;\left(\%\right)=\mathrm{SOM}\;\left(\%\right)/1.72$$

where 1.72 represents the van Bemmelen (1890) factor (conversion factor to estimate SOC from SOM, van Bemmelen, 1890). Finally, total phosphorus (TP), total potassium (TK), total iron (TFe) and total aluminium (TAl) were measured with an XRF analyser (Thermo ScientificTM Niton Portable X‐Ray Fluorescence analyser). XRF measurements were validated using a subset of samples analysed by Inductively Coupled Plasma‐Optical Emission Spectrometry (ICP‐OES) (see Figure S1).

### 2.4 DNA extraction, PCR assays and high‐throughput sequencing

Soil samples were extracted according to the saturated phosphate buffer method proposed by Taberlet et al. (2012), targeting extracellular DNA. In total, 15 mL of freshly made saturated phosphate buffer solution (Na_2_HPO_4_; 0.12 m; pH ≈ 8) was added to 15 g of soil and thoroughly mixed for 10 min. After centrifugation, the supernatant was further processed with NucleoSpin® Soil kit (Macherey‐Nagel, Düren, Germany), following the manufacturer's protocol instructions but skipping the lysis steps (Steps 1–4). The kit's lysis steps were replaced by the saturated phosphate buffer, enabling the handling of a greater soil quantity per sample (15 mg as opposed to 0.5 mg), thereby decreasing the impact of local heterogeneity. Furthermore, Zinger et al. (2016) demonstrated the reliability of extracellular DNA when examining the diversity patterns of soil bacterial communities. Negative extraction controls (saturated phosphate buffer solution) were included in every 27 extractions.

DNA concentrations of the purified DNA extracts were quantified using a multi‐mode microplate reader (Synergy™ HTX). Prior to the amplification step, DNA extracts were standardised to 5 ng/μL and diluted 100×. Field (demineralised water used for rinsing the sterilised equipment) and negative (saturated phosphate buffer solution) controls were pooled per sampled region, but their DNA concentration was not standardised nor diluted. DNA extractions were performed in a laboratory different from that used for PCR amplifications to reduce contamination risks. A 390 bp fragment of the V4 region of the 16S rRNA was amplified using the primer set 515F/806R (Apprill et al., 2015; Parada et al., 2016), including Fluidigm adaptors CS1/CS2 for multiplexing. DNA amplifications were carried out in a final volume of 12.5 μL using 2 μL of DNA extract. The amplification mixture contained 1× PCR buffer (Thermofisher), 2.5 mM of MgCl_2_ (Thermofisher), 5% of Trehalose (Fisher Scientific), 200 ng/μL of BSA (VWR), 200 μM of dNTP (VWR), 250 μM of each primer (Biolegio) and 0.08 U of Platinum Taq polymerase (Thermofisher). Cycling conditions were 94°C for 2 min, followed by 14 cycles of 94°C for 30 s, 56°C for 3 min (−1°C per cycle) and 72°C for 1 min, followed by 19 cycles of 94°C for 30 s, 42°C for 3 min and 72°C for 1 min, with a final step of 72°C for 10 min (Siegenthaler et al., 2024). Multiplexing (using i5/i7), purification of the PCR products using sparQ magnetic beads, library preparation and high‐throughput sequencing (Illumina NovaSeq 6000 SP, PE250 kit) of the amplicons were carried out by Genome Quebec (Montreal, Canada).

### 2.5 Bioinformatic analysis of sequencing data

Sequencing data were provided dereplicated by Genome Quebec, after which the QIIME 2™ software (Bolyen et al., 2019) was used for further processing. Primmer trimming was conducted using *cutadapt* (minimum‐length: 200; Martin, 2011) and *DADA‐2* (Callahan et al., 2017) was used for quality filtering, denoising, merging, chimaera removal and ASV (Amplicon Sequence Variant) construction (trunc‐len‐f: 232, trunc‐len‐r: 230, MaxEE: 2). Taxonomical assignment was performed using the SILVA database (Quast et al., 2013), followed by LULU post‐clustering curation (90% minimum match; Frøslev et al., 2017). For consistency, we used the taxonomic annotation as implemented in the most recent version of the SILVA database. However, synonyms were provided where deemed relevant (e.g. Actinobacteria synonym: Actinomycetota). Further corrections were applied to the ASV table, including blank corrections, filtering of non‐bacterial reads and tag‐switching corrections following Taberlet et al. (2018). To reduce low‐frequency noise, all taxa with less than 10 reads were removed (Alsos et al., 2016; Polling et al., 2022). Prior to data analysis, 100 rarefactions of 120,322 reads/sample were averaged to standardise read depth. Detailed information on the bioinformatic pipeline can be found in Table S1, with a summary of bioinformatic results presented in Supplementary Material 2.

### 2.6 Data processing and statistical analysis

All analyses were conducted in R version 4.3.1 (https://www.R-project.org/). All graphics were computed with the package *ggplot2* v.3.4.2 (Wickham, 2016), and all statistical analyses were performed on the rarefied samples, except if stated otherwise. We included ‘plot ID’ as a random factor in all analyses to account for spatial pseudoreplication (Chaves & Chaves, 2010). As bacterial richness and community structure strongly differed between regions (Bavarian NP vs. Veluwe NP) and forest type (deciduous vs. coniferous), we analysed each region × forest type combination separately (Tables S2 and S3). We defined soil pH as a proxy for soil acidification because it directly measures soil acidity (H^+^ concentration) and indicates the leaching of base cations (Batjes, 1995).

To identify bioindicators for moderate and extreme acidic conditions, a linear discriminant analysis (LDA) at the ASV level with soil pH as a fixed effect (i.e. explanatory variable) was performed on the combined dataset, that is including samples from both regions (Bavarian NP and Veluwe NP) and forest type (deciduous and coniferous) using the *Maaslin2* package (Mallick et al., 2021). We applied a CLR (centred log ratio) normalisation on the raw counts of the unrarefied dataset and used the ‘Linear model’ method included in the *Maaslin2* algorithm. We considered significant (*p* < 0.05) adjusted *p‐values* (Benjamini‐Hochberg correction). ASVs positively (i.e. more abundant at the highest extreme of the gradient) or negatively (i.e. more abundant at the lowest extreme of the gradient) correlated with soil pH were respectively assigned to the ‘moderate acidophile’ and ‘extreme acidophile’ categories. ASVs non‐significantly associated with soil pH were assigned to the ‘non‐categorised’ category. Bacterial ASV richness (observed number of ASVs) was computed with the *estimate_richness* function from the *phyloseq* v.1.44.0 package (McMurdie & Holmes, 2013).

The functional potential of the bacterial communities was obtained by matching the taxonomic information of the ASVs in the combined dataset with the FAPROTAX database (Louca et al., 2016). We selected functional groups with a relative abundance >0.01% related to nutrient cycling as bacteria are key agents of organic matter decomposition, nitrogen fixation and mineral weathering in temperate forest ecosystems (Lladó et al., 2017). Based on the current knowledge from the FAPROTAX database, the selected functional groups were exclusively related to the carbon and nitrogen cycles. We defined each process as described in the FAPROTAX database, including whether ASVs assigned to one functional group are also accounted for in another functional group (e.g. chemoheterotrophy). For the carbon cycle, we considered the following carbon processes: chemoheterotrophy (oxidation of organic compounds to obtain energy and carbon, including the functional groups ligninolysis, chitinolysis, xylanolysis, cellulolysis, methanol oxidation, methanotrophy, aromatic hydrocarbon degradation), aerobic chemoheterotrophy (aerobic oxidation of organic compounds to obtain energy and carbon, excluding the functional groups ligninolysis, chitinolysis, xylanolysis, cellulolysis, methanol oxidation, methanotrophy, aromatic hydrocarbon degradation), cellulolysis (hydrolysis of cellulose and hemicellulose into polysaccharides) and fermentation (breakdown of carbohydrates into glucose under anaerobic conditions). As for the nitrogen cycle, we focused on the following nitrogen processes: nitrogen fixation (biotic transformation of N_2_ from the atmosphere into NH_3_ or NH_4+_ in the soil by diazotrophs) and nitrate reduction. Here, nitrate reduction refers to both the denitrification process (transformation of NO_3−_ into N_2_) and the dissimilatory nitrate reduction process (NO_3−_ into NH_3_ or NH_4+_).

We first tested the effect of soil pH under severe acidification on bacterial richness, community composition and functional potential. We used linear mixed‐effect models for ASV richness with soil pH and forest type (deciduous vs. coniferous) as fixed effects using the *lme* function in the *nlme* v.3.1–162 package (Pinheiro et al., 2023) for each region (Bavarian NP vs. Veluwe NP) × LDA category (i.e. ASVs categorised as ‘moderate acidophile’, ‘extreme acidophile’ or ‘non‐categorised’). The effects of soil pH and forest type for each region were assessed on community composition (Hellinger‐transformed read counts) with PERMANOVA analysis (Bray–Curtis dissimilarity; 999 permutations) using the *adonis* function from the *vegan* package v.2.6–4 (Oksanen et al., 2008). We used nonmetric multidimensional scaling (NMDS) to graphically represent the relationship between soil pH and bacterial communities, as NMDS is effective at detecting underlying gradients (Ramette, 2007). We performed generalised mixed linear models (beta distribution) on the relative abundance (TSS‐transformed reads counts) of the selected functional groups using the *glmmTMB* function from the *glmmTMB* package (Brooks et al., 2017) with soil pH and forest type as fixed effects. Generalised mixed linear models were preferred over linear mixed‐effect models because the relative abundance of the functional groups did not follow a normal distribution but a beta distribution. Model assumptions were checked with the *DHARMa* v.0.4.6 package (Hartig, 2022).

Finally, we tested whether other measured soil properties than soil pH affected soil bacterial communities under severe acidification. Soil explanatory variables (pH, SOM, TP, TK, TFe, TAl, C:N ratio) did not show multicollinearity based on variance inflation factors (VIF) <10. We used linear mixed models for ASV richness and redundancy analysis (RDA) for community composition (Bray–Curtis dissimilarity of Hellinger‐transformed read counts; 999 permutations). Prior to data analysis, soil properties were scaled, and assumptions of the models were verified. RDA was selected over canonical‐correlation analysis (CCA) because detrended correspondence analysis (DCA) results showed gradient length <4 SD, meaning that linear‐based analysis was more appropriate (Ramette, 2007).

## 3 RESULTS

### 3.1 Reduction in bacterial richness and change in community structure under severe soil acidification

**FIGURE 2:**
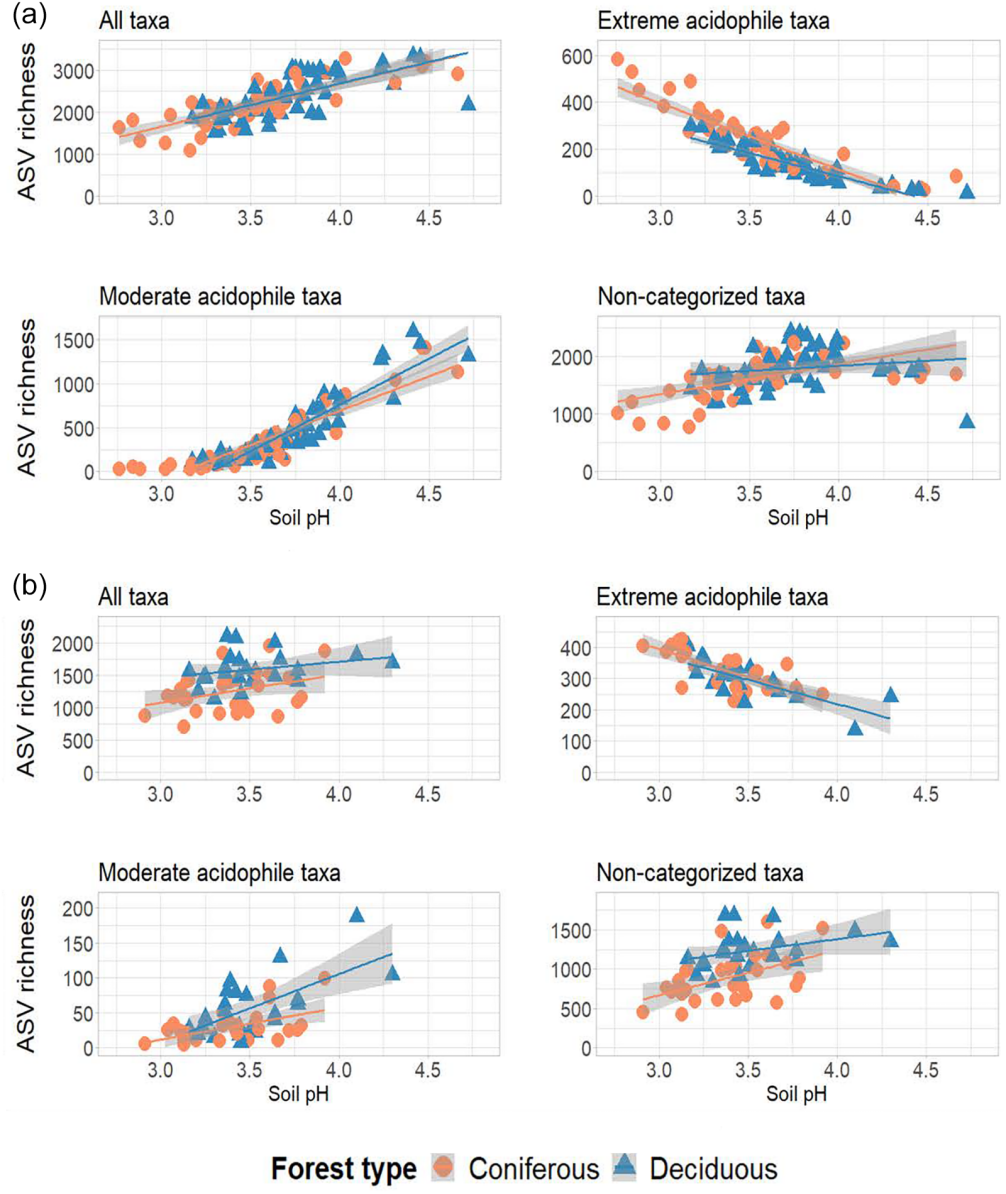
Relationships between soil pH and ASV richness for all taxa, extreme acidophile taxa (negatively correlated with soil pH), moderate acidophile taxa (positively correlated with soil pH) and non‐categorised taxa (no significant correlation with soil pH) for Bavarian NP (a) and Veluwe NP (b). Linear regression trend lines are shown per forest type (see Table S3 for details).

**FIGURE 3:**
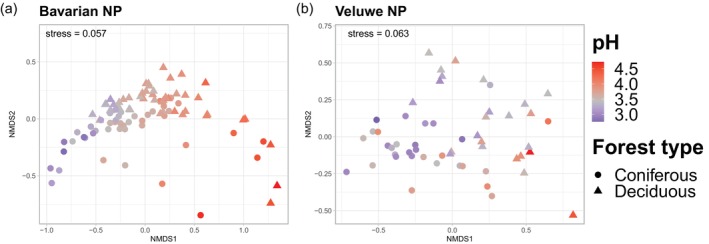
NMDS plot based on the Bray–Curtis dissimilarity of bacterial communities at the ASV level (Hellinger‐transformed reads) in Bavarian NP (a) and Veluwe NP (b). Soil samples are coloured by pH values.

Severe acidification resulted in changes in bacterial community structure and a reduction in ASV richness in both the Bavarian NP and the Veluwe NP. Bacterial richness declined along an extremely acidic soil pH gradient in both forested landscapes, showing a similar pattern in both coniferous and deciduous stands (Table S4, Figure 2). In the Bavarian NP, bacterial communities showed a clear shift along the soil pH gradient (Figure 3a; PERMANOVA: *R* ^2^ = 0.15, *F* = 16.77, *p* = 0.001). A significant but weak effect of the interaction soil pH × forest type (PERMANOVA: *R* ^2^ = 0.02, *F* = 2.81, *p* = 0.025) was observed, indicating that bacterial communities in coniferous and deciduous stands responded differently to severe acidification. Veluwe NP communities also varied with soil pH (PERMANOVA: *R* ^2^ = 0.08, *F* = 4.64, *p* = 0.001), but the shift along the soil pH gradient was less pronounced than the Bavarian NP (Figure 3b).

### 3.2 Different responses of acid‐tolerant taxa to severe acidification

**FIGURE 4:**
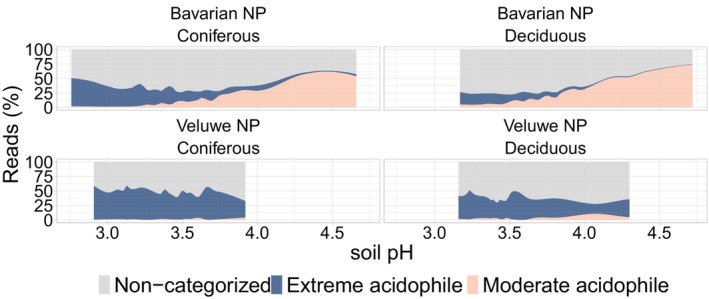
Variation of reads (%) assigned to non‐categorised taxa (not significantly correlated with soil pH), extreme acidophile taxa (negatively correlated with soil pH) and moderate acidophile taxa (positively correlated with soil pH) for each region × forest type combination along the soil pH gradient.

The extreme acidophile and moderate acidophile communities, as determined by the linear discriminant analysis, exhibited distinct responses to severe acidification (Figures 2 and 4). In the extreme acidophile community, Acidothermaceae (13.58 ± 3.87%), Acidobacteria GP1 (12.37 ± 5.43%) and Solibacteraceae (7.71 ± 4.34%) were the predominant families, while in the moderate acidophile community, the families Xanthobacteraceae (13.36 ± 8.30%), Solirubrobacteraceae (12.37 ± 15.29%) and Subgroup_2 (8.44 ± 7.35%) dominated (Figure S2). Additional information on the different acid‐tolerant groups can be found in Supplementary Material 2. The observed loss of richness was primarily due to the loss of both moderate acidophile and non‐categorised (i.e. taxa not classified as acid‐tolerant) in the Bavarian NP and the loss of non‐categorised taxa only in the Veluwe NP (Table S4, Figure 2). Forest type only influenced the relationship between soil pH and the richness of extreme acidophile and non‐categorised taxa in the Bavarian NP (Table S4, Figure 2.A). As acidity increased, coniferous stands gained more extreme acidophile taxa but experienced a greater decline in non‐categorised taxa compared to deciduous stands.

When taxon abundances were grouped per linear discriminant analysis category, a clear shift in community structure and taxonomical profiles with soil pH was observed (Figure 4 and S2). In the Veluwe NP, the proportion of moderate acidophile taxa was low, even at the highest end of the pH gradient (pH >3.5). The proportion of extreme acidophile taxa displayed distinct patterns across regions, remaining stable along the soil pH gradient in the Veluwe NP but increasing under more severe acidification in the Bavarian NP. Within each region, coniferous and deciduous stands exhibited similar patterns, although coniferous stands had a higher proportion of extreme acidophile taxa than deciduous stands across the pH range. A brief summary of the linear discriminant analysis results can be found in Supplementary Material 2.

### 3.3 Shifts in functional groups with severe acidification

**FIGURE 5:**
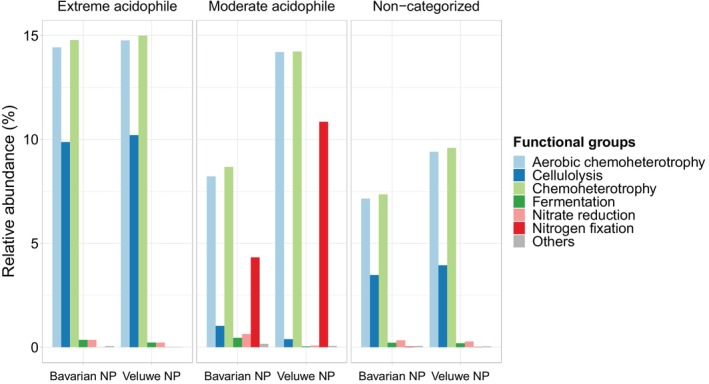
Mean relative abundance of the most abundant nitrogen and carbon‐related functional groups (relative abundance >0.01%) for extreme acidophile taxa (negatively correlated with soil pH), moderate acidophile taxa (positively correlated with soil pH) and non‐categorised taxa (not significantly correlated with soil pH) for each region. The remaining functional groups are grouped under the category ‘Others’. The proportion of reads that could not be assigned to a functional group has been removed to enhance the visual representation.

Severe acidification consistently promoted carbon‐related functions while decreasing or eliminating nitrogen‐related functions for both the Bavarian NP and the Veluwe NP (Figure 5, Table 1). In the Bavarian NP, all functional groups, except fermentation and nitrate reduction, significantly varied with soil pH, while in the Veluwe NP, only cellulolysis was significantly associated with soil pH variation (Table 1). Extreme acidophile and moderate acidophile communities showed distinct putative functional profiles, and these functional profiles were approximately consistent for both forested landscapes in The Netherlands and Germany (Figure 5). The moderate acidophile community was characterised by a higher nitrogen fixation potential compared to the extreme acidophile community (Bavarian NP: 4.32 ± 3.35% vs. 0.0004 ± 0.003%; Veluwe NP: 10.85 ± 3.64 vs. 0.004 ± 0.008%) and a lower cellulolysis potential (Bavarian NP: 1.02 ± 0.76% vs. 9.87 ± 2.29%; Veluwe NP: 0.38 ± 0.87% vs. 10.20 ± 2.20%). Forest type significantly influenced the response of some functional groups to soil pH variation in both the Bavarian NP and the Veluwe NP (Table 1). Specifically, fermentation and nitrate reduction in Bavarian NP and chemoheterotrophy and aerobic chemoheterotrophy in Veluwe NP were lower in deciduous stands compared to coniferous stands. These functional groups also showed forest type specific responses, with potential activity increasing along the soil pH gradient for deciduous stands while remaining stable for coniferous stands. Overall, the variation in relative abundances of the studied functional groups (Table 1) was well explained for cellulolysis, where soil pH and forest type alone could explain 66% (Bavarian NP) and 50% (Veluwe NP) of the variation. Other functional groups had lower explained variation.

**TABLE 1:** Results of generalised linear mixed models for soil pH, forest type (deciduous vs. coniferous) and soil pH × forest type on the relative abundance of different functional groups involved in carbon and nitrogen cycle per region.

	*R* ^2^m	*R* ^2^c	Soil pH	Forest type	Soil pH × forest type
Bavarian NP					
Chemoheterotrophy	0.21	0.55	−0.29 (−2.77)**	−0.29 (−0.51)	0.06 (0.40)
Aerobic chemoheterotrophy	0.22	0.54	−0.31 (−2.71)**	−0.05 (−0.09)	−0.001 (−0.006)
Cellulolysis	0.66	0.73	−0.73 (−7.13)***	0.20 (0.35)	−0.10 (−0.59)
Fermentation	0.17	0. 43	0.17 (0.79)	−2.99 (−2.61)**	0.80 (2.59)**
Nitrogen fixation	0.32	0.57	0.68 (4.51)***	0.97 (1.25)	−0.23 (−1.11)
Nitrate reduction	0.29	0.52	0.23 (1.77)	−2.12 (−3.11)**	0.56 (3.04)**
Veluwe NP					
Chemoheterotrophy	0.27	0.52	−0.20 (−1.02)	−1.95 (−2.16)*	0.50 (1.93)*
Aerobic chemoheterotrophy	0.26	0.56	−0.24 (−1.20)	−2.07 (−2.19)*	0.54 (1.97)*
Cellulolysis	0.50	0.72	−0.52 (−3.07)**	−1.41 (−1.51)	0.31 (1.17)
Fermentation	0.02	0.55	0.36 (0.60)	2.18 (0.74)	−0.60 (−0.70)
Nitrogen fixation	0.27	0.65	0.65 (1.77)	−0.20 (−0.11)	0.18 (0.36)
Nitrate reduction	0.03	0.54	0.32 (0.63)	0.79 (0.32)	−0.18 (−0.26)

*Note*: Regression estimates (*z* value) are indicated for each fixed effect. Bold regression estimates refer to significant *p‐values* (*<0.05; **<0.01; ***<0.001). *R* ^2^m: adjusted *R* ^2^ for fixed effects. *R* ^2^c: adjusted *R* ^2^ for both fixed and random effects.

### 3.4 Response of bacterial communities to soil properties under severe acidification

We assessed the relationship between bacterial richness and bacterial communities with soil properties (pH, SOM, TP, TK, TFe, TAl, C:N ratio) under severe acidification using linear mixed‐effect models and redundancy analysis (RDA), respectively. Soil pH was the only predictor (*p* < 0.01) of bacterial richness in the Bavarian NP. ASV richness in the Veluwe NP was positively associated with total soil iron (TFe) (*p* < 0.01) in deciduous stands and both soil pH (*p* < 0.01) and total aluminium (TAl) (*p* < 0.01) in coniferous stands. The linear mixed‐effect models explained a larger proportion of variation in bacterial ASV richness in Bavarian NP compared to Veluwe NP (49%–65% vs. 34%–35%, Table 2). Bacterial communities in Bavarian NP were significantly (*p* < 0.001) associated with soil pH, while SOM was significantly (*p* < 0.05) associated with bacterial communities in the coniferous stands of the Veluwe NP (Table 3). The RDA model for the deciduous stands in the Veluwe NP was not significant (*p =* 0.14), indicating that none of the tested soil biochemical properties significantly explained variation in community composition. The mean ± standard deviation of each biochemical property is indicated in Table S5.

**TABLE 2:** Results of linear mixed‐effect models for soil biochemical properties measured on bacterial ASV richness per region × forest type combination.

	pH	SOM	TP	TK	TFe	TAl	C:N ratio	*R* ^2^m	*R* ^2^c
Bavarian NP									
Coniferous	230**	−87	83	−9	216	−143	49	0.65	0.78
Deciduous	168*	5	−117	3	−115	401	72	0.49	0.66
Veluwe NP									
Coniferous	205**	−14	71	−345	−238	1778*	49	0.35	0.80
Deciduous	35	76	−133	−335	948*	41	−123	0.34	0.39

*Note*: Scaled regression estimates are shown for each soil biochemical property. Bold estimates refer to significant p‐values (*<0.05; **<0.01; ***<0.001). *R* ^2^m: adjusted *R* ^2^ for fixed effects. *R* ^2^c: adjusted *R* ^2^ for both fixed and random effects.

**TABLE 3:** Results of redundancy analysis models for soil biochemical properties measured on soil bacterial communities at the ASV level (Hellinger‐transformed reads).

	Adjusted *R* ^2^	Variable	df	Variance	*F*	*p*‐value
Bavarian NP						
Coniferous	0.20***	**pH**	**1**	**0.0295**	**3.2665**	**0.009**
		SOM	1	0.0140	1.5496	0.444
		TP	1	0.0172	1.9041	0.154
		TK	1	0.0128	1.4205	0.533
		Fe	1	0.0137	1.5232	0.312
		TAl	1	0.0166	1.8437	0.350
		C:N ratio	1	0.0010	1.0578	0.777
		Residuals	37	0.3337		
Deciduous	0.20***	**pH**	**1**	**0.0315**	**4.0466**	**0.001**
		SOM	1	0.0082	1.0516	0.561
		TP	1	0.0093	1.1954	0.489
		TK	1	0.0122	1.5637	0.452
		TFe	1	0.0073	1.9311	0.448
		TAl	1	0.0090	1.1545	0.291
		C:N ratio	1	0.0107	1.3771	0.139
		Residuals	40	0.3117		
Veluwe NP						
Coniferous	0.10*	pH	1	0.0169	1.4712	0.075
		**SOM**	**1**	**0.0224**	**1.9537**	**0.031**
		TP	1	0.0165	1.4350	0.411
		TK	1	0.0167	1.4586	0.148
		TFe	1	0.0146	1.2740	0.135
		TAl	1	0.0120	1.0482	0.206
		C:N ratio	1	0.0076	0.6625	0.920
		Residuals	22	0.2524		
Deciduous	0.14	pH	1	0.0157	1.0501	0.925
		SOM	1	0.0143	0.9548	0.372
		TP	1	0.0206	1.3751	0.193
		TK	1	0.0128	0.8580	0.248
		TFe	1	0.0212	1.4149	0.366
		TAl	1	0.0156	1.0436	0.155
		C:N ratio	1	0.0161	1.0776	0.219
		Residuals	16	0.2396		

*Note*: For each model, adjusted *R* ^2^ is indicated. Bold adjusted *R* ^2^ values refer to significant *p*‐values of the full model (*<0.05; **<0.01; ***<0.001). The degree of freedom (df), variance, *F*‐statistic (*F*) and *p*‐values are indicated for each soil biochemical variable. Significant variables (*p* < 0.05) are highlighted in bold.

## 4 DISCUSSION

### 4.1 Ongoing acidification in very acidic soils decreases bacterial diversity

Acidification of soil is an important driver of soil bacterial communities, resulting in the selection of taxa based on their capacity to withstand the extremely acid environment, aluminium toxicity, direct physiological stress and low nutrient availability (Da Mota et al., 2008; Fierer & Jackson, 2006; Lauber et al., 2009; Norman & Barrett, 2016; Rousk et al., 2010). For the first time in natural conditions, we showed that bacterial communities continue to change along a declining soil pH gradient, even under severe pH levels, as recorded in our study areas. It has been postulated that in extremely acidic soils (pH <4.5), bacterial communities are already highly specialised and have limited responses to further acidification (Rousk et al., 2009). Our study demonstrates that shifts in bacterial communities correlate with soil pH in already severely acidic forest soils due to human‐caused nitrogen atmospheric deposition. The results of this study contrast with our original hypothesis (i) (see Section 1) because earlier researchers had not studied bacterial communities in such severely acidic habitats. Given that other European temperate forests are experiencing similar acidity levels, our findings underscore the importance of including these habitats in conservation efforts (Abdullah et al., 2024; Šantrůčková et al., 2019).

Further, we observed a sustained loss of bacterial diversity alongside a shift in community structure under the severely acidic habitats studied here. This shift favoured extremely acid‐tolerant families like Acidobacteria GP1, Acidothermaceae and Solibacteraceae over mildly acidic‐tolerant families such as Xanthobacteraceae and Solirubacteraceae (Bárta et al., 2017; Carvalho & Castillo, 2018). These changes in the tolerance levels of bacteria under severely acidic soils may be linked to the physiological traits of acid‐stress tolerant species and their ability to thrive in the oligotrophic and carbon‐limited environments of extremely acidic soils. Bacteria specialised in degrading carbon‐recalcitrant compounds and organic matter have a major advantage in these conditions compared to bacteria involved in the nitrogen cycle (Bárta et al., 2017; Choma et al., 2020; Wan et al., 2022). This advantage leads to changes in putative functional soil profiles. As soil pH decreases, the availability of carbon to bacteria also decreases while organic matter accumulates (Evans et al., 2012). To survive in nutrient‐ and carbon‐limited environments such as extremely acidic soils, the selected bacteria show a higher capacity for carbon mineralisation and degradation of complex organic substrates (Hagh‐Doust et al., 2023).

To further investigate these changes in putative functional soil profiles as soil pH decreases, we used the FAPROTAX database to link metabarcoding‐based taxonomic identifications directly to putative functional profiles. We found that severe soil acidification promotes carbon‐related functions and suppresses nitrogen‐related functions (Table 1, Figure 5). Our results corroborate previous research using various methods such as putative functional approaches, quantitative PCR analysis (qPCR), metagenomics and enzymatic activity (Choma et al., 2020; Hagh‐Doust et al., 2023; Wan et al., 2022).

Increase in cellulolytic activity with acidification has been consistently reported in various ecosystems such as agricultural fields (Rothenberg et al., 2022; Wan et al., 2022; Wang et al., 2019), watersheds (Sridhar et al., 2022), peatlands (Fenner et al., 2005) or forests (Du et al., 2014). In contrast, while alterations in several carbon‐related functions have been noted in other studies, cellulolytic activity remains unchanged, likely due to site‐specific conditions (Hagh‐Doust et al., 2023; Waldrop et al., 2004). This finding aligns with our finding of a shift towards extremely acidic‐tolerant taxa at the end of the gradient, with these taxa being typically specialised in breaking down carbon‐recalcitrant compounds such as cellulose (Choma et al., 2020). Solely relying on details about the relative abundance of acidophilic taxa engaged in carbon degradation may be challenging to understand the potential impact on the carbon cycle. In specific scenarios, such as organic nitrogen deposition, the enhancement of cellulolysis, and carbon compound breakdown in general, could result in carbon loss, potentially shifting forest systems from a carbon sink to a carbon source (Du et al., 2014; Wang et al., 2019). However, past research indicated that, beyond shifts in community structure, soil acidification adversely affects microbial respiration, biomass and enzymatic activity, ultimately slowing down the decomposition rate and resulting in soil carbon sequestration (Chuman et al., 2021; Hagedorn et al., 2003). It is not possible to determine which scenarios apply to the forests we studied, as the necessary data were unavailable.

Additionally, nitrogen fixation and nitrate reduction processes are typically suppressed under acidification, as observed in similar temperate forests (Jaiswal et al., 2018; Lin et al., 2022; Shang et al., 2023; Truu et al., 2020; Zhou et al., 2016). The loss of nitrogen fixation can be explained by either the induced aluminium toxicity inhibiting nodulation of symbiotic bacteria such as Rhizobiales (Bakari et al., 2020; Bordeleau & Prévost, 1994; He et al., 2012) or the competition for resources (Lauber et al., 2009; Tahovská et al., 2020; Wan et al., 2022). For the Veluwe NP, the ongoing high nitrogen atmospheric deposition eases the N‐limitation typical of terrestrial ecosystems (Kopáček et al., 2013). Therefore, investment in such genes may not be essential (Lin et al., 2022; Zheng et al., 2017). Since functions may be attributed only to a limited portion of the bacterial community (based on current scientific knowledge), making assumptions about the putative functional profiles of the community as a whole remains somewhat tenuous. Consequently, alterations in putative functional profiles do not, by definition, consistently align with changes in activity (Baldrian et al., 2012). Yet, FAPROTAX‐derived annotations can provide us with some initial indications of the implications of reduced taxonomic diversity for functional redundancy (Labouyrie et al., 2023; Yang et al., 2022) under extreme soil acidification. Additional analysis, such as metagenomics, could be conducted to test our hypotheses further and include more nitrogen processes that were omitted here, such as nitrification, which is a key indicator of nitrogen losses in temperate forests (Tahovská et al., 2020).

### 4.2 Similar community responses across forest types

Bacterial communities in deciduous and coniferous stands exhibited similar responses to severe acidification within each region (Figures 3 and 4), contracting the hypothesis that bacterial communities in coniferous stands would be less sensitive to severe acidification than those in deciduous stands. We found, in general, a higher relative abundance of extreme acidophile taxa in coniferous stands, which is consistent with other studies in temperate forests, where soil acidification induced comparable shifts in bacterial communities (Choma et al., 2020; Shen et al., 2021). For instance, the richer deciduous litter results in higher turnover and faster decomposition, with soil conditions favouring copiotrophic microorganisms (Prescott & Grayston, 2013). These studies, like ours, focused on topsoil, where the link between below‐ and aboveground processes is the strongest (Delgado‐Baquerizo et al., 2017). However, Xu et al. (2023) demonstrated that including deep soil layers reveals more clearly that forest type impacts bacterial community characteristics and functions. This suggests the need to include these lower soil profile layers in future acidification studies.

While severe acidification yielded similar patterns of bacterial communities across forest regions, the Veluwe NP exhibited a weaker response than those in the Bavarian NP. This disparity may possibly be attributed to regional differences in nitrogen deposition, leading to varying acidification rates of forest national parks in Europe, coupled with the lower buffering capacity of soils in the sandy soils of the Veluwe NP (Skidmore et al., 2024). As a result of the ongoing acidification pressure and the homogeneity in soil substrate (e.g. sandy oligotrophic soil) across the Veluwe NP soil, conditions are relatively homogeneous, giving rise to more homogeneous and less varied bacterial communities. Furthermore, bacterial communities in the Veluwe NP were significantly associated with localised soil with higher organic matter content in the otherwise alluvial sandy soils. In contrast, soils in the Bavarian NP were exclusively correlated with soil pH. Soil organic matter content in the Veluwe NP is a primary energy source and a proxy for soil organic carbon quantity, which are key factors in shaping bacterial communities (Delgado‐Baquerizo et al., 2016; Ding et al., 2015). Dissolved organic carbon (DOC), resulting from soil organic matter decomposition, is a direct source of energy for microorganisms (Kaiser & Kalbitz, 2012). DOC has been shown to correlate with soil organic matter content in temperate forests (Lee et al., 2023). Furthermore, the availability of DOC is influenced by soil pH, so as soil pH decreases (e.g. due to soil acidification), the availability of carbon to microorganisms also decreases (Ekström et al., 2011; Evans et al., 2012; Pschenyckyj et al., 2020). Our findings confirm our third hypothesis, showing that in extremely acidic and poor soils like in the Veluwe NP, other biochemical parameters than soil pH, such as soil organic matter, may limit bacterial communities.

## 5 CONCLUSION

European soils are rapidly degrading, with soil biodiversity under pressure across approximately 56% of the total European land cover (Gardi et al., 2013). Here, we demonstrate that forest bacterial biodiversity and functions continue to degrade with increasing acidification, even in soils with severe pH levels (pH <4.5). Soil bacterial communities, which serve as bioindicators of forest health due to their essential role in plant–soil interactions (Herrera Paredes & Lebeis, 2016), highlight these findings as an early warning signal of stress on forest health and biodiversity. Forest soil acidity continues to worsen due to nitrogen compound deposition in regional European forested areas, including sensitive Natura 2000 areas where pH levels can be as low as the acidity of domestic vinegar (Skidmore et al., 2024). Soil protection is an integral part of European Union soil governance, including initiatives such as the European Green Deal, the EU Biodiversity Strategy and the EU Soil Strategy for 2030 (European Commission, 2021). Our findings underscore the urgency of including these severely acidified forests in these conservation efforts.

## AUTHOR CONTRIBUTIONS

**Mélody Rousseau**: Conceptualization, Methodology, Investigation, Data collection, Formal Analysis, Visualisation, Validation, Writing—original draft. **Andrew K. Skidmore**: Conceptualization, Validation, Supervision, Resources, Project Administration, Funding acquisition, Writing—review and editing. **Andjin Siegenthaler**: Methodology, Data collection, Formal analysis, Validation, Writing—review and editing. **G. Arjen de Groot**: Conceptualization, Methodology, Validation, Supervision, Resources, Writing—review and editing. **Ivo Laros**: Formal analysis, Validation, Writing—review and editing.

## FUNDING INFORMATION

This work was supported by the European Research Council (grant 397 agreement ID 834709, H2020‐EU.1.1).

## Supporting information


Supplementary Material 1.

Supplementary Material 2.

## Data Availability

The data that support the findings of this study are available in the Supplementary Material of this article.

## References

[ejss70005-bib-0001] Abdullah, H. , Skidmore, A. K. , Siegenthaler, A. , Adiningrat, D. P. , Duan, Y. , & Rousseau, M. (2024). Temperate forest soil pH accurately quantified with image spectroscopy. Remote Sensing Applications: Society and Environment, 34, 101161. 10.1016/j.rsase.2024.101161

[ejss70005-bib-0002] Ahrends, B. , Fortmann, H. , & Meesenburg, H. (2022). The influence of tree species on the recovery of forest soils from acidification in Lower Saxony, Germany. Soil Systems, 6(2), 40. 10.3390/SOILSYSTEMS6020040

[ejss70005-bib-0003] Alsos, I. G. , Sjögren, P. , Edwards, M. E. , Landvik, J. Y. , Gielly, L. , Forwick, M. , Coissac, E. , Brown, A. G. , Jakobsen, L. V. , Føreid, M. K. , & Pedersen, M. W. (2016). Sedimentary ancient DNA from Lake Skartjørna, Svalbard: Assessing the resilience of arctic flora to Holocene climate change. The Holocene, 26(4), 627–642. 10.1177/0959683615612563

[ejss70005-bib-0004] Apprill, A. , Mcnally, S. , Parsons, R. , & Weber, L. (2015). Minor revision to V4 region SSU rRNA 806R gene primer greatly increases detection of SAR11 bacterioplankton. Aquatic Microbial Ecology, 75, 129–137. 10.3354/AME01753

[ejss70005-bib-0005] Augusto, L. , Ranger, J. , Binkley, D. , & Rothe, A. (2002). Impact of several common tree species of European temperate forests on soil fertility. Annals of Forest Science, 59(3), 233–253. 10.1051/forest:2002020

[ejss70005-bib-0006] Bakari, R. , Mungai, N. , Thuita, M. , & Masso, C. (2020). Impact of soil acidity and liming on soybean (*Glycine max*) nodulation and nitrogen fixation in Kenyan soils. Acta Agriculturae Scandinavica Section B Soil and Plant Science, 70(8), 667–678. 10.1080/09064710.2020.1833976

[ejss70005-bib-0007] Baker, N. J. , Pilotto, F. , Jourdan, J. , Beudert, B. , & Haase, P. (2021). Recovery from air pollution and subsequent acidification masks the effects of climate change on a freshwater macroinvertebrate community. Science of the Total Environment, 758, 143685. 10.1016/j.scitotenv.2020.143685 33288265

[ejss70005-bib-0008] Baldrian, P. , Kolaiřík, M. , Štursová, M. , Kopecký, J. , Valášková, V. , Větrovský, T. , Žifčáková, L. , Šnajdr, J. , Rídl, J. , Vlček, Č. , & Voříšková, J. (2012). Active and total microbial communities in forest soil are largely different and highly stratified during decomposition. The ISME Journal, 6, 248–258. 10.1038/ismej.2011.95 21776033 PMC3260513

[ejss70005-bib-0009] Bani, A. , Pioli, S. , Ventura, M. , Panzacchi, P. , Borruso, L. , Tognetti, R. , Tonon, G. , & Brusetti, L. (2018). The role of microbial community in the decomposition of leaf litter and deadwood. Applied Soil Ecology, 126, 75–84. 10.1016/j.apsoil.2018.02.017

[ejss70005-bib-0010] Barak, P. , Jobe, B. O. , Krueger, A. R. , Peterson, L. A. , & Laird, D. A. (1997). Effects of long‐term soil acidification due to nitrogen fertilizer inputs in Wisconsin. Plant and Soil, 197, 61–69. 10.1023/A:1004297607070

[ejss70005-bib-0011] Bárta, J. , Tahovská, K. , Šantrůčková, H. , & Oulehle, F. (2017). Microbial communities with distinct denitrification potential in spruce and beech soils differing in nitrate leaching. Scientific Reports, 7, 9738. 10.1038/s41598-017-08554-1 28851897 PMC5575336

[ejss70005-bib-0012] Bässler, C. , Seifert, L. , & Müller, J. (2015). The BIOKLIM project in the National Park Bavarian Forest: Lessons from a biodiversity survey. Silva Gabreta, 21(1), 81–93.

[ejss70005-bib-0013] Batjes, N. H. (1995). A global data set of soil pH properties. Technical Paper 27. International Soil Reference and Information Centre (ISRIC). https://www.isric.org/sites/default/files/ISRIC_TechPap27.pdf

[ejss70005-bib-0014] Black, C. A. (1965). Methods of soil analysis. Part 1. Physical and mineralogical properties, including statistics of measurement and sampling. Part 2. Chemical and microbiological properties. Agronomy Series no. 9, ASA.

[ejss70005-bib-0015] Bolyen, E. , Rideout, J. R. , Dillon, M. R. , Bokulich, N. A. , Abnet, C. C. , Al‐Ghalith, G. A. , Alexander, H. , Alm, E. J. , Arumugam, M. , & Asnicar, F. (2019). Reproducible, interactive, scalable and extensible microbiome data science using QIIME 2. Nature Biotechnology, 37, 852–857. 10.1038/s41587-019-0209-9 PMC7015180 31341288

[ejss70005-bib-0016] Bordeleau, L. M. , & Prévost, D. (1994). Nodulation and nitrogen fixation in extreme environments. Plant and Soil, 161, 115–125. 10.1007/BF02183092

[ejss70005-bib-0017] Brooks, M. E. , Kristensen, K. , van Benthem, K. J. , Magnusson, A. , Berg, C. W. , Nielsen, A. , Skaug, H. J. , Maechler, M. , & Bolker, B. M. (2017). glmmTMB balances speed and flexibility among packages for zero‐inflated generalized linear mixed modeling. The R Journal, 9(2), 378–400. 10.32614/RJ-2017-066

[ejss70005-bib-0018] Buresova, A. , Kopecky, J. , Hrdinkova, V. , Kamenik, Z. , Omelka, M. , & Sagova‐Mareckova, M. (2019). Succession of microbial decomposers is determined by litter type, but site conditions drive decomposition rates. Applied and Environmental Microbiology, 85(24), e01760. 10.1128/AEM.01760-19 31604765 PMC6881812

[ejss70005-bib-0019] Callahan, B. J. , McMurdie, P. J. , & Holmes, S. P. (2017). Exact sequence variants should replace operational taxonomic units in marker‐gene data analysis. ISME Journal, 11, 2369–2643. 10.1038/ismej.2017.119 PMC5702726 28731476

[ejss70005-bib-0020] Carvalho, S. D. , & Castillo, J. A. (2018). Influence of light on plant–phyllosphere interaction. Frontiers in Plant Science, 9, 291–298. 10.3389/fpls.2018.01482 30369938 PMC6194327

[ejss70005-bib-0021] Chaves, L. F. , & Chaves, L. F. (2010). An entomologist guide to demystify pseudoreplication: Data analysis of field studies with design constraints. Journal of Medical Entomology, 47(3), 291–298. 10.1093/JMEDENT/47.1.291 20496574

[ejss70005-bib-0022] Chen, Y. , Yin, S. , Shao, Y. , & Zhang, K. (2022). Soil bacteria are more sensitive than fungi in response to nitrogen and phosphorus enrichment. Frontiers in Microbiology, 13, 999385. 10.3389/fmicb.2022.999385 36212871 PMC9537584

[ejss70005-bib-0023] Choma, M. , Tahovská, K. , Kaštovská, E. , Bárta, J. , Růžek, M. , & Oulehle, F. (2020). Bacteria but not fungi respond to soil acidification rapidly and consistently in both a spruce and beech forest. FEMS Microbiology Ecology, 96(10), fiaa174. 10.1093/femsec/fiaa174 32815987

[ejss70005-bib-0024] Chuman, T. , Oulehle, F. , Zajícová, K. , & Hruška, J. (2021). The legacy of acidic deposition controls soil organic carbon pools in temperate forests across The Czech Republic. European Journal of Soil Science, 72, 1780–1801. 10.1111/EJSS.13073

[ejss70005-bib-0025] Da Mota, F. F. , Gomes, E. A. , Marriel, I. E. , & Sledin, L. (2008). Bacterial and fungal communities in bulk soil and rhizospheres of aluminum‐tolerant and aluminum‐sensitive maize (*Zea mays L*.) lines cultivated in unlimed and limed Cerrado soil. Journal of Microbiology and Biotechnology, 18(5), 805–814. 18633275

[ejss70005-bib-0026] de Hoop, E. , Brouwers, H. J. H. , Buijs, S. L. , Egberts, L. , van Gerrevink, M. J. , de Ruiter, M. C. , & Veraverbeke, S. (2022). Multi‐stakeholder analysis of fire risk reduction in a densely populated area in The Netherlands: A case‐study in the Veluwe area. Environmental Research Letters, 17, 095011. 10.1088/1748-9326/AC8B97

[ejss70005-bib-0027] Delgado‐Baquerizo, M. , Eldridge, D. J. , Ochoa, V. , Gozalo, B. , Singh, B. K. , & Maestre, F. T. (2017). Soil microbial communities drive the resistance of ecosystem multifunctionality to global change in drylands across the globe. Ecology Letters, 20(10), 1295–1305. 10.1111/ELE.12826 28921861

[ejss70005-bib-0028] Delgado‐Baquerizo, M. , Maestre, F. T. , Reich, P. B. , Trivedi, P. , Osanai, Y. , Liu, Y. R. , Hamonts, K. , Jeffries, T. C. , & Singh, B. K. (2016). Carbon content and climate variability drive global soil bacterial diversity patterns. Ecological Monographs, 86(3), 373–390. 10.1002/ECM.1216

[ejss70005-bib-0029] Dentener, F. , Drevet, J. , Lamarque, J. F. , Bey, I. , Eickhout, B. , Fiore, A. M. , Hauglustaine, D. , Horowitz, L. W. , Krol, M. , Kulshrestha, U. C. , Lawrence, M. , Galy‐Lacaux, C. , Rast, S. , Shindell, D. , Stevenson, D. , Van Noije, T. , Atherton, C. , Bell, N. , Bergman, D. , … Wild, O. (2006). Nitrogen and sulfur deposition on regional and global scales: A multimodel evaluation. Global Biogeochemical Cycles, 20(4), 1–21. 10.1029/2005GB002672

[ejss70005-bib-0030] Ding, J. , Zhang, Y. , Wang, M. , Sun, X. , Cong, J. , Deng, Y. , Lu, H. , Yuan, T. , Van Nostrand, J. D. , Li, D. , Zhou, J. , & Yang, Y. (2015). Soil organic matter quantity and quality shape microbial community compositions of subtropical broadleaved forests. Molecular Ecology, 24(20), 5175–5185. 10.1111/MEC.13384 26363284

[ejss70005-bib-0031] Du, Y. , Guo, P. , Liu, J. , Wang, C. , Yang, N. , & Jiao, Z. (2014). Different types of nitrogen deposition show variable effects on the soil carbon cycle process of temperate forests. Global Change Biology, 20(10), 3222–3228. 10.1111/GCB.12555 24615991

[ejss70005-bib-0032] EC . (2004). The European soil database distribution version 2.0. European Commission and the European Soil Bureau Network, CD‐ROM, EUR 19945 EN.

[ejss70005-bib-0033] EEA . (2020). European Union emission inventory report 1990–2018 under the UNECE Convention on Long‐Range Transboundary Air Pollution (LRTAP) (EEA Report 05/2020).

[ejss70005-bib-0034] EEA . (2022). Emissions of the main air pollutants in Europe. European Environment Agency. Retrieved 25 November 2022 from https://www.eea.europa.eu/ims/emissions-of-the-main-air

[ejss70005-bib-0035] Ekström, S. M. , Kritzberg, E. S. , Kleja, D. B. , Larsson, N. , Nilsson, P. A. , Graneli, W. , & Bergkvist, B. (2011). Effect of acid deposition on quantity and quality of dissolved organic matter in soil–water. Environmental Science & Technology, 45(11), 4733–4739. 10.1021/es104126f 21528901

[ejss70005-bib-0036] EMEP . (2024). Gridded SO_X_ data. Centre on Emission Inventories and Projections. Retrieved 29 May 2024 from https://www.ceip.at/the-emep-grid/gridded-emissions/sox

[ejss70005-bib-0037] European Commission . (2021). Communication from the commission to the European parliament, the council, the European economic and social committee and the committee of the regions – EU Soil Strategy for 2030: reaping the benefits of healthy soils for people, food, nature and climate. COM (2021) 699 final of 17.11.2021, p. 1 ff.

[ejss70005-bib-0038] Evans, C. D. , Jones, T. G. , Burden, A. , Ostle, N. , Zieliński, P. , Cooper, M. D. A. , Peacock, M. , Clark, J. M. , Oulehle, F. , Cooper, D. , & Freeman, C. (2012). Acidity controls on dissolved organic carbon mobility in organic soils. Global Change Biology, 18(11), 3317–3331. 10.1111/j.1365-2486.2012.02794.x

[ejss70005-bib-0039] Fagerli, H. , Tsyro, S. , Rolstad Denby, B. , Nyíri, Á. , Gauss, M. , Simpson, D. , Wind, P. , Benedictow, A. , Eiof Jonson, J. , Klein, H. , Schulz, M. , Griesfeller, J. , Aas, W. , Hjellbrekke, A.‐G. , Solberg, S. , Matthew Platt, S. , Fiebig, M. , Espen Yttri, K. , Olav Rud, R. , … Holubova Smejkalova, A. (2017). Transboundary particulate matter, photo‐oxidants, acidifying and eutrophying components. https://emep.int/publ/reports/2017/EMEP_Status_Report_1_2017.pdf

[ejss70005-bib-0040] Fenner, N. , Freeman, C. , & Reynolds, B. (2005). Hydrological effects on the diversity of phenolic degrading bacteria in a peatland: Implications for carbon cycling. Soil Biology and Biochemistry, 37(7), 1277–1287. 10.1016/j.soilbio.2004.11.024

[ejss70005-bib-0041] Fierer, N. , & Jackson, R. B. (2006). The diversity and biogeography of soil bacterial communities. Proceedings of the National Academy of Sciences of the United States of America, 103(3), 626–631. 10.1073/pnas.0507535103 16407148 PMC1334650

[ejss70005-bib-0111] Forstner, S. J. , Wechselberger, V. , Stecher, S. , Müller, S. , Keiblinger, K. M. , Wanek, W. , Schleppi, P. , Gundersen, P. , Tatzber, M. , Gerzabek, M. H. , & Zechmeister‐Boltenstern, S. (2019). Resistant soil microbial communities show signs of increasing phosphorus limitation in two temperate forests after long‐term nitrogen addition. Frontiers in Forests and Global Change, 2, 488417. 10.3389/ffgc.2019.00073

[ejss70005-bib-0042] Frøslev, T. G. , Kjøller, R. , Bruun, H. H. , Ejrnæs, R. , Brunbjerg, A. K. , Pietroni, C. , & Hansen, A. J. (2017). Algorithm for post‐clustering curation of DNA amplicon data yields reliable biodiversity estimates. Nature Communications, 8, 1188. 10.1038/s41467-017-01312-x PMC5662604 29084957

[ejss70005-bib-0043] Gardi, C. , Jeffery, S. , & Saltelli, A. (2013). An estimate of potential threats levels to soil biodiversity in EU. Global Change Biology, 19(5), 1538–1548. 10.1111/GCB.12159 23505125

[ejss70005-bib-0044] Goedickemeier, I. , Wildi, O. , & Kienast, F. (1997). Sampling for vegetation survey: Some properties of a GIS‐based stratification compared to other statistical sampling methods. JSTOR, 12(1), 43–50. https://www.jstor.org/stable/43461187

[ejss70005-bib-0045] Hagedorn, F. , Spinnler, D. , & Siegwolf, R. (2003). Increased N deposition retards mineralization of old soil organic matter. Soil Biology & Biochemistry, 35, 1683–1692. 10.1016/j.soilbio.2003.08.015

[ejss70005-bib-0046] Hagh‐Doust, N. , Mikryukov, V. , Anslan, S. , Bahram, M. , Puusepp, R. , Dulya, O. , & Tedersoo, L. (2023). Effects of nitrogen deposition on carbon and nutrient cycling along a natural soil acidity gradient as revealed by metagenomics. New Phytologist, 238(6), 2607–2620. 10.1111/NPH.18897 36949609

[ejss70005-bib-0047] Handley, W. (1954). Mull and mor formations in relation to forest soils . Bull. 23, Forestry Commission, pp. 115. https://cdn.forestresearch.gov.uk/1954/04/fcbu023.pdf

[ejss70005-bib-0048] Hartig, F. (2022). DHARMa: Residual Diagnostics for Hierarchical (Multi‐Level/Mixed) Regression Models. R package version 0.4.

[ejss70005-bib-0049] Hein, L. (2011). Economic benefits generated by protected areas: The case of the Hoge Veluwe forest, The Netherlands. Ecology and Society, 16(2), 13. https://www.jstor.org/stable/26268887

[ejss70005-bib-0112] He, G. , Lin, J. , Liu, Q. , Zhang, J. , & Wu, J. (2012). The effects of aluminum stress on bacterial community diversity in acidic red soils by polymerase chain reaction (PCR)‐amplified restriction fragment length polymorphism. African Journal of Microbiology Research, 6(15), 3707–3715. 10.5897/ajmr12.347

[ejss70005-bib-0050] Herrera Paredes, S. , & Lebeis, S. L. (2016). Giving back to the community: Microbial mechanisms of plant–soil interactions. Functional Ecology, 30(7), 1043–1052. 10.1111/1365-2435.12684

[ejss70005-bib-0051] Heurich, M. , Beudert, B. , Rall, H. , & Křenová, Z. (2010). National parks as model regions for interdisciplinary long‐term ecological research: The Bavarian Forest and Šumavá national parks underway to transboundary ecosystem research. In F. Müller , C. Baessler , H. Schubert , & S. Klotz (Eds.), Long‐term ecological research (pp. 327–344). Springer. 10.1007/978-90-481-8782-9_23

[ejss70005-bib-0052] Hornung, M. (1985). Acidification of soils by trees and forests. Soil Use and Management, 1(1), 24–27. 10.1111/j.1475-2743.1985.tb00648.x

[ejss70005-bib-0053] Hu, Z. , Delgado‐Baquerizo, M. , Fanin, N. , Chen, X. , Zhou, Y. , Du, G. , Hu, F. , Jiang, L. , Hu, S. , & Liu, M. (2024). Nutrient‐induced acidification modulates soil biodiversity‐function relationships. Nature Communications, 15, 2858. 10.1038/s41467-024-47323-3 PMC10991381 38570522

[ejss70005-bib-0054] Jaiswal, S. K. , Naamala, J. , & Dakora, F. D. (2018). Nature and mechanisms of aluminium toxicity, tolerance and amelioration in symbiotic legumes and rhizobia. Biology and Fertility of Soils, 54, 309–318. 10.1007/S00374-018-1262-0 31258230 PMC6560468

[ejss70005-bib-0055] Kaiser, K. , & Kalbitz, K. (2012). Cycling downwards – Dissolved organic matter in soils. Soil Biology and Biochemestry, 52, 29–32. 10.1016/J.SOILBIO.2012.04.002

[ejss70005-bib-0056] Kaiser, K. , Wemheuer, B. , Korolkow, V. , Wemheuer, F. , Nacke, H. , Schöning, I. , Schrumpf, M. , & Daniel, R. (2016). Driving forces of soil bacterial community structure, diversity, and function in temperate grasslands and forests. Scientific Reports, 6, 33696. 10.1038/srep33696 27650273 PMC5030646

[ejss70005-bib-0057] Kopáček, J. , Cosby, J. C. , Evans, D. E. , Hruška, J. , Moldan, F. , Oulehle, F. , Šantrůčková, S. , Tahovská, K. , & Wright, R. F. (2013). Nitrogen, organic carbon and sulphur cycling in terrestrial ecosystems: Linking nitrogen saturation to carbon limitation of soil microbial processes. Biogeochemistry, 155, 33–51. 10.1007/S10533-013-9892-7

[ejss70005-bib-0058] Labouyrie, M. , Ballabio, C. , Romero, F. , Panagos, P. , Jones, A. , Schmid, M. W. , Mikryukov, V. , Dulya, O. , Tedersoo, L. , Bahram, M. , Lugato, E. , van der Heijden, M. G. A. , & Orgiazzi, A. (2023). Patterns in soil microbial diversity across Europe. Nature Communications, 14, 1–21. 10.1038/s41467-023-37937-4 PMC10250377 37291086

[ejss70005-bib-0059] Lajtha, K. , & Jones, J. (2013). Trends in cation, nitrogen, sulfate and hydrogen ion concentrations in precipitation in the United States and Europe from 1978 to 2010: A new look at an old problem. Biogeochemistry, 116, 303–334. 10.1007/S10533-013-9860-2

[ejss70005-bib-0060] Lauber, C. L. , Hamady, M. , Knight, R. , & Fierer, N. (2009). Pyrosequencing‐based assessment of soil pH as a predictor of soil bacterial community structure at the continental scale. Applied and Environmental Microbiology, 75(15), 5111–5120. 10.1128/AEM.00335-09 19502440 PMC2725504

[ejss70005-bib-0061] Lee, J. , Oh, Y. , Lee, S. T. , Seo, Y. O. , Yun, J. , Yang, Y. , Kim, J. , Zhuang, Q. , & Kang, H. (2023). Soil organic carbon is a key determinant of CH_4_ sink in global forest soils. Nature Communications, 14(1), 1–8. 10.1038/s41467-023-38905-8 PMC10229549 37253779

[ejss70005-bib-0062] Lin, S. , Liu, Z. , Wang, Y. , Li, J. , Wang, G. , Ye, J. , Wang, H. , & He, H. (2022). Soil metagenomic analysis on changes of functional genes and microorganisms involved in nitrogen‐cycle processes of acidified tea soils. Frontiers in Plant Science, 13, 1–13. 10.3389/fpls.2022.998178 PMC9614370 36311106

[ejss70005-bib-0063] Lladó, S. , López‐Mondéjar, R. , & Baldrian, P. (2017). Forest soil bacteria: Diversity, involvement in ecosystem processes, and response to global change. Microbiology and Molecular Biology Reviews, 81, 1–27. 10.1128/mmbr.00063-16 PMC5485800 28404790

[ejss70005-bib-0064] Lladó, S. , López‐Mondéjar, R. , & Baldrian, P. (2018). Drivers of microbial community structure in forest soils. Applied Microbiology and Biotechnology, 102, 4331–4338. 10.1007/s00253-018-8950-4 29600493

[ejss70005-bib-0065] Louca, S. , Parfrey, L. W. , & Doebeli, M. (2016). Decoupling function and taxonomy in the global ocean microbiome. Science, 353(6305), 1272–1277. 10.1126/science.aaf4507 27634532

[ejss70005-bib-0066] Mallick, H. , Rahnavard, A. , McIver, L. J. , Ma, S. , Zhang, Y. , Nguyen, L. H. , Tickle, T. L. , Weingart, G. , Ren, B. , Schwager, E. H. , Chatterjee, S. , Thompson, K. N. , Wilkinson, J. E. , Subramanian, A. , Lu, Y. , Waldron, L. , Paulson, J. N. , Franzosa, E. A. , Bravo, H. C. , & Huttenhower, C. (2021). Multivariable association discovery in population‐scale meta‐omics studies. PLoS Computational Biology, 17(11), e1009442. 10.1371/journal.pcbi.1009442 34784344 PMC8714082

[ejss70005-bib-0067] Martin, M. (2011). Cutadapt removes adapter sequences from high‐throughput sequencing reads. EMBnet.Journal, 17(1), 10–12. 10.14806/EJ.17.1.200

[ejss70005-bib-0068] McMurdie, P. J. , & Holmes, S. (2013). *Phyloseq*: An R package for reproducible interactive analysis and graphics of microbiome census data. PLoS One, 8(4), e61217. 10.1371/JOURNAL.PONE.0061217 23630581 PMC3632530

[ejss70005-bib-0069] Mol, G. , Vriend, S. P. , & van Gaans, P. F. M. (2003). Feldspar weathering as the key to understanding soil acidification monitoring data; a study of acid sandy soils in The Netherlands. Chemical Geology, 202(3–4), 417–441. 10.1016/J.CHEMGEO.2002.07.001

[ejss70005-bib-0070] Nacke, H. , Thürmer, A. , Wollherr, A. , Will, C. , Hodac, L. , Herold, N. , Schöning, I. , Schrumpf, M. , & Daniel, R. (2011). Pyrosequencing‐based assessment of bacterial community structure along different management types in German forest and grassland soils. PLoS One, 6(2), e17000. 10.1371/journal.pone.0017000 21359220 PMC3040199

[ejss70005-bib-0071] Norman, J. S. , & Barrett, J. E. (2016). Substrate availability drives spatial patterns in richness of ammonia‐oxidizing bacteria and archaea in temperate forest soils. Soil Biology and Biochemistry, 94, 169–172. 10.1016/j.soilbio.2015.11.015

[ejss70005-bib-0072] Oksanen, J. , Kindt, R. , Legendre, P. , O'Hara, B. , Simpson, G. L. , Solymos, P. , Stevens, M. H. H. , & Wagner, H. (2008). *vegan*: community ecology package (R package version 2.6‐4). https://CRAN.R-project.org/package=vegan

[ejss70005-bib-0073] Parada, A. E. , Needham, D. M. , & Fuhrman, J. A. (2016). Every base matters: Assessing small subunit rRNA primers for marine microbiomes with mock communities, time series and global field samples. Environmental Microbiology, 18(5), 1403–1414. 10.1111/1462-2920.13023 26271760

[ejss70005-bib-0074] Pingali, P. (2012). Green revolution: Impacts, limits, and the path ahead. Proceedings of the National Academy of Sciences, 109(31), 12302–12308. 10.1073/PNAS.0912953109 PMC3411969 22826253

[ejss70005-bib-0075] Pinheiro, J. , Bates, D. , & R Core Team . (2023). *nlme*: linear and nonlinear mixed effects models (R package version 3.1–162). https://CRAN.R-project.org/package=nlme

[ejss70005-bib-0076] Polling, M. , Sin, M. , de Weger, L. A. , Speksnijder, A. G. C. L. , Koenders, M. J. F. , de Boer, H. , & Gravendeel, B. (2022). DNA metabarcoding using nrITS2 provides highly qualitative and quantitative results for airborne pollen monitoring. Science of the Total Environment, 806, 150468. 10.1016/j.scitotenv.2021.150468 34583071 PMC8651626

[ejss70005-bib-0077] Posch, M. , Duan, L. , Reinds, G. J. , & Zhao, Y. (2015). Critical loads of nitrogen and sulphur to avert acidification and eutrophication in Europe and China. Landscape Ecology, 30(3), 487–499. 10.1007/S10980-014-0123-Y

[ejss70005-bib-0078] Prescott, C. , & Grayston, S. (2013). Tree species influence on microbial communities in litter and soil: Current knowledge and research needs. Forest Ecology and Management, 309, 19–27. 10.1016/j.foreco.2013.02.034

[ejss70005-bib-0079] Pschenyckyj, C. M. , Clark, J. M. , Shaw, L. J. , Griffiths, R. I. , & Evans, C. D. (2020). Effects of acidity on dissolved organic carbon in organic soil extracts, pore water and surface litters. Science of the Total Environment, 703, 135585. 10.1016/J.SCITOTENV.2019.135585 31767326

[ejss70005-bib-0080] Quast, C. , Pruesse, E. , Yilmaz, P. , Gerken, J. , Schweer, T. , Yarza, P. , Peplies, J. , & Glöckner, F. O. (2013). The SILVA ribosomal RNA gene database project: Improved data processing and web‐based tools. Nucleic Acids Research, 41, D590–D596. 10.1093/NAR/GKS1219 23193283 PMC3531112

[ejss70005-bib-0081] Ramette, A. (2007). Multivariate analyses in microbial ecology. FEMS Microbiology Ecology, 62(2), 142–160. 10.1111/j.1574-6941.2007.00375.x 17892477 PMC2121141

[ejss70005-bib-0082] Ramirez, K. , Craine, J. , & Fierer, N. (2012). Consistent effects of nitrogen amendments on soil microbial communities and processes across biomes. Global Change Biology, 18, 1918–1927. 10.1111/j.1365-2486.2012.02639.x

[ejss70005-bib-0083] Rothenberg, D. O. N. , Abbas, F. , Mei, X. , Yan, C. , Zeng, Z. , Mo, X. , Chen, S. , Zhang, L. , & Huang, Y. (2022). Metabarcoding of organic tea (*Camellia sinensis L*.) chronosequence plots elucidates soil acidification‐induced shifts in microbial community structure and putative function. Applied Soil Ecology, 178, 104580. 10.1016/J.APSOIL.2022.104580

[ejss70005-bib-0084] Rousk, J. , Bååth, E. , Brookes, P. C. , Lauber, C. L. , Lozupone, C. , Caporaso, J. G. , Knight, R. , Fierer, N. , Rousk, J. , Ba, E. , Caporaso, J. G. , Knight, R. , & Fierer, N. (2010). Soil bacterial and fungal communities across a pH gradient in an arable soil. The ISME Journal, 4, 1340–1351. 10.1038/ismej.2010.58 20445636

[ejss70005-bib-0085] Rousk, J. , Brookes, P. C. , & Bååth, E. (2009). Contrasting soil pH effects on fungal and bacterial growth suggest functional redundancy in carbon mineralization. Applied and Environmental Microbiology, 75(6), 1589–1596. 10.1128/AEM.02775-08 19151179 PMC2655475

[ejss70005-bib-0086] Šantrůčková, H. , Cienciala, E. , Kaňa, J. , & Kopáček, J. (2019). The chemical composition of forest soils and their degree of acidity in Central Europe. Science of the Total Environment, 687, 96–103. 10.1016/j.scitotenv.2019.06.078 31203012

[ejss70005-bib-0087] Shang, S. , Song, M. , Wang, C. , Dou, X. , Wang, J. , Liu, F. , Zhu, C. , & Wang, S. (2023). Decrease of nitrogen cycle gene abundance and promotion of soil microbial‐N saturation restrain increases in N_2_O emissions in a temperate forest with long‐term nitrogen addition. Chemosphere, 338, 139378. 10.1016/j.chemosphere.2023.139378 37419152

[ejss70005-bib-0088] Shen, Y. , Tian, D. , Hou, J. , Wang, J. , Zhang, R. , Li, Z. , Chen, X. , Wei, X. , Zhang, X. Y. , He, Y. , & Niu, S. (2021). Forest soil acidification consistently reduces litter decomposition irrespective of nutrient availability and litter type. Functional Ecology, 35(12), 2753–2762. 10.1111/1365-2435.13925

[ejss70005-bib-0089] Siegenthaler, A. , Skidmore, A. K. , de Groot, G. A. , Laros, I. , Rousseau, M. , & Duan, Y. (2024). Temperate tree microbiomes: Divergent soil and phyllosphere microbial communities share few but dominant taxa. Plant and Soil, 496, 319–340. 10.1007/s11104-023-06364-1

[ejss70005-bib-0090] Simpson, D. , Andersson, C. , Christensen, J. H. , Engardt, M. , Geels, C. , Nyiri, A. , Posch, M. , Soares, J. , Sofiev, M. , Wind, P. , & Langner, J. (2024). Impacts of climate and emission changes on nitrogen deposition in Europe: A multi‐model study. Atmospheric Chemistry and Physics, 14(13), 6995–7017. 10.5194/acp-14-6995-2014

[ejss70005-bib-0091] Skidmore, A. K. , Abdullah, H. , Siengathler, A. , Adiningrat, D. P. , Rousseau, M. , Duan, Y. , & Torres‐Rodriguez, A. (2024). Forest soils further acidify in core Natura 200 areas amongst unaware government policy. Ecological Indicators, 159, 111621. 10.1016/j.ecolind.2024.111621

[ejss70005-bib-0092] Sridhar, B. , Wilhelm, R. C. , Debenport, S. J. , Fahey, T. J. , Buckley, D. H. , & Goodale, C. L. (2022). Microbial community shifts correspond with suppression of decomposition 25 years after liming of acidic forest soils. Global Change Biology, 28(18), 5399–5415. 10.1111/gcb.16321 35770362

[ejss70005-bib-0093] Taberlet, P. , Bonin, A. , Zinger, L. , & Coissac, E. (2018). Environmental DNA: For biodiversity research and monitoring. Oxford University Press. 10.1093/oso/9780198767220.001.0001

[ejss70005-bib-0094] Taberlet, P. , Coissac, E. , Pompanon, F. , Brochmann, C. , & Willerslev, E. (2012). Towards next‐generation biodiversity assessment using DNA metabarcoding. Molecular Ecology, 21(8), 2045–2050. 10.1111/j.1365-294X.2012.05470.x 22486824

[ejss70005-bib-0095] Tahovská, K. , Choma, M. , Kaštovská, E. , Oulehle, F. , Bárta, J. , Šantrůčková, H. , & Moldan, F. (2020). Positive response of soil microbes to long‐term nitrogen input in spruce forest: Results from Gårdsjön whole‐catchment N‐addition experiment. Soil Biology and Biochemistry, 143, 107732. 10.1016/j.soilbio.2020.107732

[ejss70005-bib-0096] Truu, M. , Nõlvak, H. , Ostonen, I. , Oopkaup, K. , Maddison, M. , Ligi, T. , Espenberg, M. , Uri, V. , Mander, Ü. , & Truu, J. (2020). Soil bacterial and archaeal communities and their potential to perform N‐cycling processes in soils of boreal forests growing on well‐drained peat. Frontiers in Microbiology, 11, 3112. 10.3389/FMICB.2020.591358 PMC7744593 33343531

[ejss70005-bib-0097] Urbanová, M. , Šnajdr, J. , & Baldrian, P. (2015). Composition of fungal and bacterial communities in forest litter and soil is largely determined by dominant trees. Soil Biology and Biochemistry, 84, 53–64. 10.1016/j.soilbio.2015.02.011

[ejss70005-bib-0098] van Bemmelen, J. M. (1890). Ueber die Bestimmung des Wassers, des Humus, des Mangans, im Ackerboden. Landwirtsch Versuchsstat, 37, 279–290. https://edepot.wur.nl/211282

[ejss70005-bib-0099] van der Knaap, W. O. , van Leeuwen, J. F. N. , Fahse, L. , Szidat, S. , Studer, T. , Baumann, J. , Heurich, M. , & Tinner, W. (2019). Vegetation and disturbance history of the Bavarian Forest National Park, Germany. Vegetation History and Archaeobotany, 29(2), 277–295. 10.1007/S00334-019-00742-5

[ejss70005-bib-0100] van Reeuwijk, L. P. (2002). Procedure for soil analysis (6th ed.). International Soil Reference and Information Centre (ISRIC). https://www.isric.org/sites/default/files/ISRIC_TechPap09.pdf

[ejss70005-bib-0101] Vestreng, V. , Myhre, G. , Fagerli, H. , Reis, S. , & Tarrasón, L. (2007). Twenty‐five years of continuous sulphur dioxide emission reduction in Europe. Atmospheric Chemistry and Physics, 7, 3663–3681. www.atmos-chem-phys.net/7/3663/2007/

[ejss70005-bib-0102] Waldrop, M. P. , Zak, D. R. , Sinsabaugh, R. L. , Gallo, M. , & Lauber, C. (2004). Nitrogen deposition modifies soil carbon storage through changes in microbial enzymatic activity. Ecological Applications, 14(4), 1172–1177. 10.1890/03-5120

[ejss70005-bib-0103] Wan, W. , Tan, J. , Wang, Y. , Qin, Y. , He, H. , Wu, H. , Zuo, W. , & He, D. (2022). Responses of the rhizosphere bacterial community in acidic crop soil to pH: Changes in diversity, composition, interaction, and function. Science of the Total Environment, 700, 134418. 10.1016/j.scitotenv.2019.134418 31629269

[ejss70005-bib-0104] Wang, C.‐Y. , Zhou, X. , Guo, D. , Zhao, J.‐H. , Yan, L. , Feng, G.‐Z. , Gao, Q. , Yu, H. , & Zhao, L.‐P. (2019). Soil pH is the primary factor driving the distribution and function of microorganisms in farmland soils in northeastern China. Annals of Microbiology, 69, 1461–1473. 10.1007/s13213-019-01529-9

[ejss70005-bib-0105] Wickham, H. (2016). ggplot2: Elegant graphics for data analysis. Springer‐Verlag. https://ggplot2.tidyverse.org

[ejss70005-bib-0106] Xu, Z. , Hu, Z. , Jiao, S. , Bell, S. M. , Xu, Q. , Ma, L. , & Chen, J. (2023). Depth‐dependent effects of tree species identity on soil microbial community characteristics and multifunctionality. Science of the Total Environment, 878, 162972. 10.1016/j.scitotenv.2023.162972 36958562

[ejss70005-bib-0107] Yang, Z. , Peng, C. , Cao, H. , Song, J. , Gong, B. , Li, L. , Wang, L. , He, Y. , Liang, M. , Lin, J. , & Lu, L. (2022). Microbial functional assemblages predicted by the FAPROTAX analysis are impacted by physicochemical properties, but C, N and S cycling genes are not in mangrove soil in the Beibu gulf, China. Ecological Indicators, 139, 10887. 10.1016/J.ECOLIND.2022.108887

[ejss70005-bib-0108] Zheng, M. , Zhang, W. , Luo, Y. , Mori, T. , Mao, Q. , Wang, S. , Huang, J. , Lu, X. , & Mo, J. (2017). Different responses of asymbiotic nitrogen fixation to nitrogen addition between disturbed and rehabilitated subtropical forests. Science of the Total Environment, 601–602, 1505–1512. 10.1016/J.SCITOTENV.2017.06.036 28605868

[ejss70005-bib-0109] Zhou, J. , Deng, Y. , Shen, L. , Wen, C. , Yan, Q. , Ning, D. , Qin, Y. , Xue, K. , Wu, L. , He, Z. , Voordeckers, J. W. , Van Nostrand, J. D. , Buzzard, V. , Michaletz, S. T. , Enquist, B. J. , Weiser, M. D. , Kaspari, M. , Waide, R. , Yang, Y. , … Brown, J. H. (2016). Temperature mediates continental‐scale diversity of microbes in forest soils. Nature Communications, 7, 12083. 10.1038/ncomms12083 PMC4935970 27377774

[ejss70005-bib-0110] Zinger, L. , Chave, J. , Coissac, E. , Iribar, A. , Louisanna, E. , Manzi, S. , Schilling, V. , Schimann, H. , Sommeria‐Klein, G. , & Taberlet, P. (2016). Extracellular DNA extraction is a fast, cheap and reliable alternative for multi‐taxa surveys based on soil DNA. Soil Biology & Biochemistry, 96, 16–19. 10.1016/j.soilbio.2016.01.008

