# Supplementary material for: Further reduction in soil bacterial diversity under severe acidification in European temperate forests
Source: Eur J Soil Sci. 2024 Nov 8;75(6):e70005. doi: 10.1111/ejss.70005 (PMC11579971; doi:10.1111/ejss.70005)
Supplement: Supplementary file 1 — Supplementary Material 1. [file EJSS-75-e70005-s002.docx]

# **Supplementary material 1**

**Table S1.** Bioinformatic pipeline for 16S metabarcoding data. Steps 2 and 3 were conducted in QIIME 2™ and steps 4 to 10 in R version 4.3.0. Non-default parameters are provided.

| **Step** | **Package** | **Parameters** | **Reads** | **ASVs** | **Samples** |
| --- | --- | --- | --- | --- | --- |
| 1. Demultiplexing |  |  | 173,874,448 (F/R) |  | 493 |
| 2. Primer trimming | cutadapt | minimum-length: 200 | 168,445,114 (F/R) |  | 493 |
| 3. Denoising and merging | dada2 | trunc-len-f: 232  trunc-len-r: 230  MaxEE: 2 | 116,120,466 | 149,143 | 493 |
| 4. Post-clustering curation | LULU | minimum_match: 90%  minimum_relative_cooccurence: 0.95 | 116,120,466 | 113,598 | 493 |
| 5. Blank correction | Custom R-script | Removal criteria: max reads in blanks ≥ max reads in samples | 112,363,185 | 112,923 | 478* |
| 6. Non-bacterial read filter | Custom R-script | Removal of reads with domain ≠ bacteria | 79,405,492 | 104,499 | 478 |
| 7. Tag-switching | Custom R-script | Tag-switching threshold: 0.003% | 79,400,076 | 104,499 | 478 |
| 8. Sample selection | Custom R-script | Exclusion of samples that are not pertinent to this study | 37,861,061 | 77,870 | 147 |
| 9. Low frequency noise filtering | Custom R-script | Removal of ASVs with < 10 reads in total | 37,757,007 | 42,947 | 147 |
| 10. Rarefaction | Vegan | Subsample size: 120,322 reads | 17,689,536 | 42,947 | 147 |

* Removal of 6 positive control samples and 9 negative control samples.

**Table S2.** Results of PERMANOVA analysis for region (Bavarian NP vs. Veluwe NP), forest type (deciduous vs. coniferous), and region x forest type on soil bacterial communities at the ASV level (Bray-Curtis dissimilarity of Hellinger-transformed read counts; 999 permutations). The degree of freedom (df), sum of squares, R^2^, F-statistic (F), and *p-value*s are indicated for each variable.

| **Variable** | **df** | **Sum of Squares** | **R^2^** | **F** | ***p-value*** |
| --- | --- | --- | --- | --- | --- |
| Region | 1 | 9.190 | 0.23 | 47.1432 | 0.001 |
| Forest type | 1 | 1.094 | 0.03 | 5.6100 | 0.016 |
| Region x forest type | 1 | 0.978 | 0.02 | 5.0163 | 0.037 |
| Residuals | 143 | 27.875 | 0.71 |  |  |

**Table S3.** Results of ANOVA analysis for region (Bavarian NP vs. Veluwe NP), forest type (deciduous vs. coniferous), and region x forest type on bacterial ASV richness. The degree of freedom (df), sum of squares, F-statistic (F), and *p-values* are indicated for each variable.

| **Variable** | **df** | **Sum of Squares** | **F** | ***p-value*** |
| --- | --- | --- | --- | --- |
| Region | 1 | 8.962 | 175.74 | < 0.001 |
| Forest type | 1 | 0.941 | 18.45 | < 0.001 |
| Region x forest type | 1 | 0.235 | 4.61 | 0.0335 |
| Residual | 143 | 0.051 |  |  |

**Table S4.** Results of linear mixed models for soil pH, forest type (deciduous vs. coniferous) and soil pH x forest type on bacterial ASV richness for each region (Bavarian NP vs. Veluwe NP) x LDA category. Regression estimates(degree of freedom) are indicated for each fixed effect. Bold regression estimates refer to significant p-values (* < 0.05; ** < 0.01; *** < 0.001). R^2^m: adjusted R^2^ for fixed effects. R^2^c: adjusted R^2^ for both fixed and random effects.

|  | *R^2^m* | *R^2^c* | soil pH | forest type | soil pH x forest type |
| --- | --- | --- | --- | --- | --- |
| Bavarian NP |  |  |  |  |  |
| All taxa | 0.48 | 0.69 | **929(60)***** | 470(29) | -118(60) |
| Extreme acidophile taxa | 0.81 | 0.90 | **-287(60)***** | **-496(29)***** | **119(60)***** |
| Moderate acidophile taxa | 0.80 | 0.90 | **769(60)***** | -574(29) | 153(60) |
| Non-categorized taxa | 0.16 | 0.60 | **413(60)**** | **1655(29)*** | -**419(60)**** |
| Veluwe NP |  |  |  |  |  |
| All taxa | 0.36 | 0.62 | **545(34)**** | 1274(16) | -284(34) |
| Extreme acidophile taxa | 0.57 | 0.67 | **-195(34)***** | -176(16) | 49(34) |
| Moderate acidophile taxa | 0.45 | 0.61 | **56(34)**** | -97(16) | 33(34) |
| Non-categorized taxa | 0.46 | 0.69 | **692(34)***** | 1584(16) | -378(34) |

**Table S5.** Mean ± standard deviation (sd) of each soil biochemical property measured per region x forest type.

|  | **Bavarian NP** | | **Veluwe NP** | |
| --- | --- | --- | --- | --- |
|  | *Coniferous* | *Deciduous* | *Coniferous* | *Deciduous* |
| **pH** | 3.54 ± 0.41 | 3.75 ± 0.33 | 3.37 ± 0.26 | 3.50 ± 0.27 |
| **SOM** | 19.09 ± 10.74 | 12.09 ± 4.74 | 12.37 ± 7.86 | 6.67 ± 3.05 |
| **TP** | 644 ± 138 | 621 ± 88 | 406 ± 64 | 458 ± 109 |
| **TK** | 4,973 ± 2,185 | 4,130 ± 2,583 | 1,592 ± 569 | 2,227 ± 411 |
| **TFe** | 11,818 ± 5,353 | 16,380 ± 2,586 | 3,468 ± 1,019 | 4,258 ± 580 |
| **TAl** | 11,227 ± 5,085 | 15,665 ± 3,499 | 2,494 ± 843 | 3,643 ± 967 |
| **C:N ratio** | 19.64 ± 3.81 | 16.90 ± 4,74 | 22.37 ± 5.17 | 16.11 ± 2.38 |

**
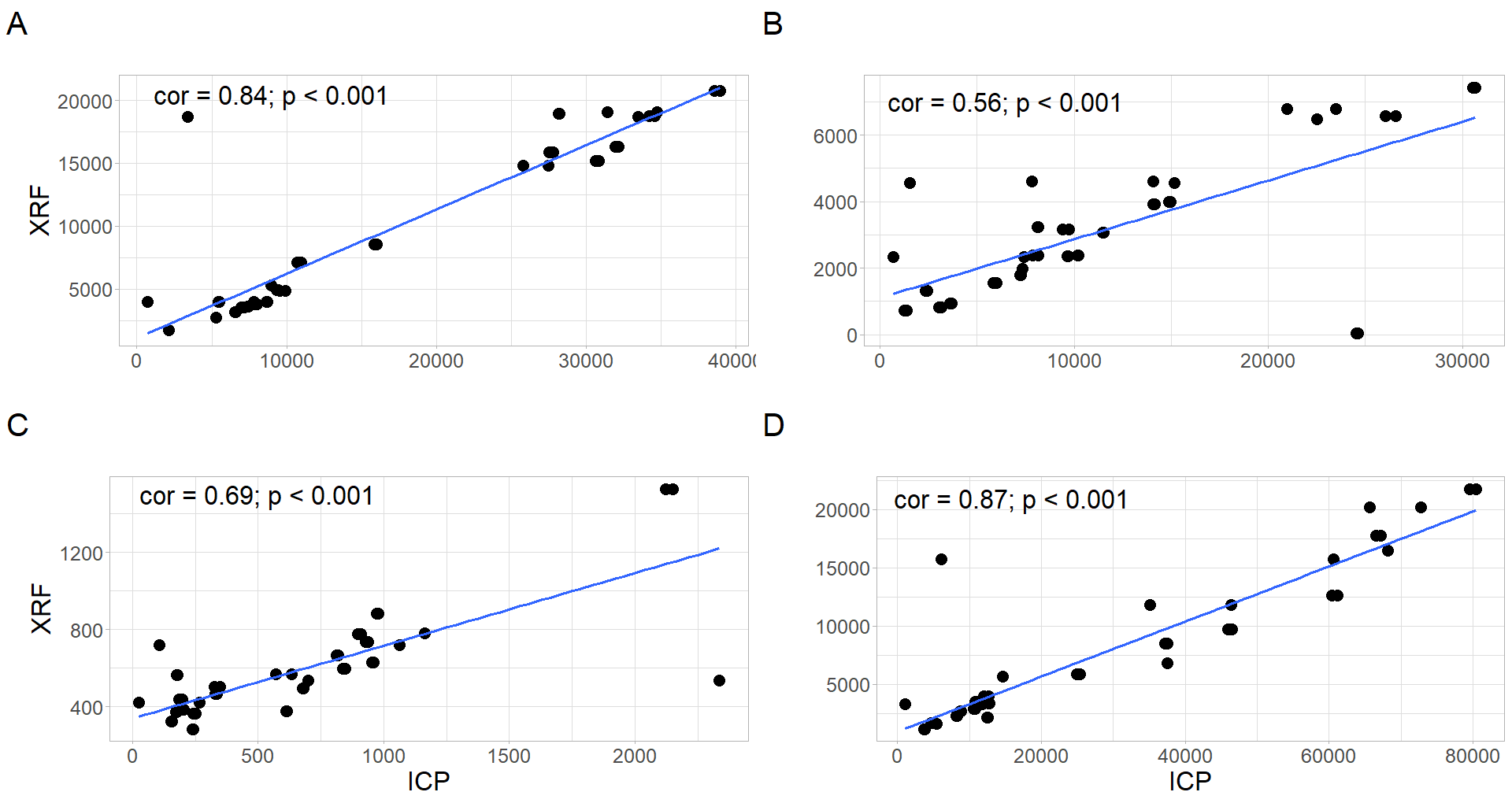
**

**Figure S1.** Scatter plots and correlation results between XRF and ICP-OES measurements for total iron (A), total potassium (B), total phosphorus (C), and total aluminium (D).


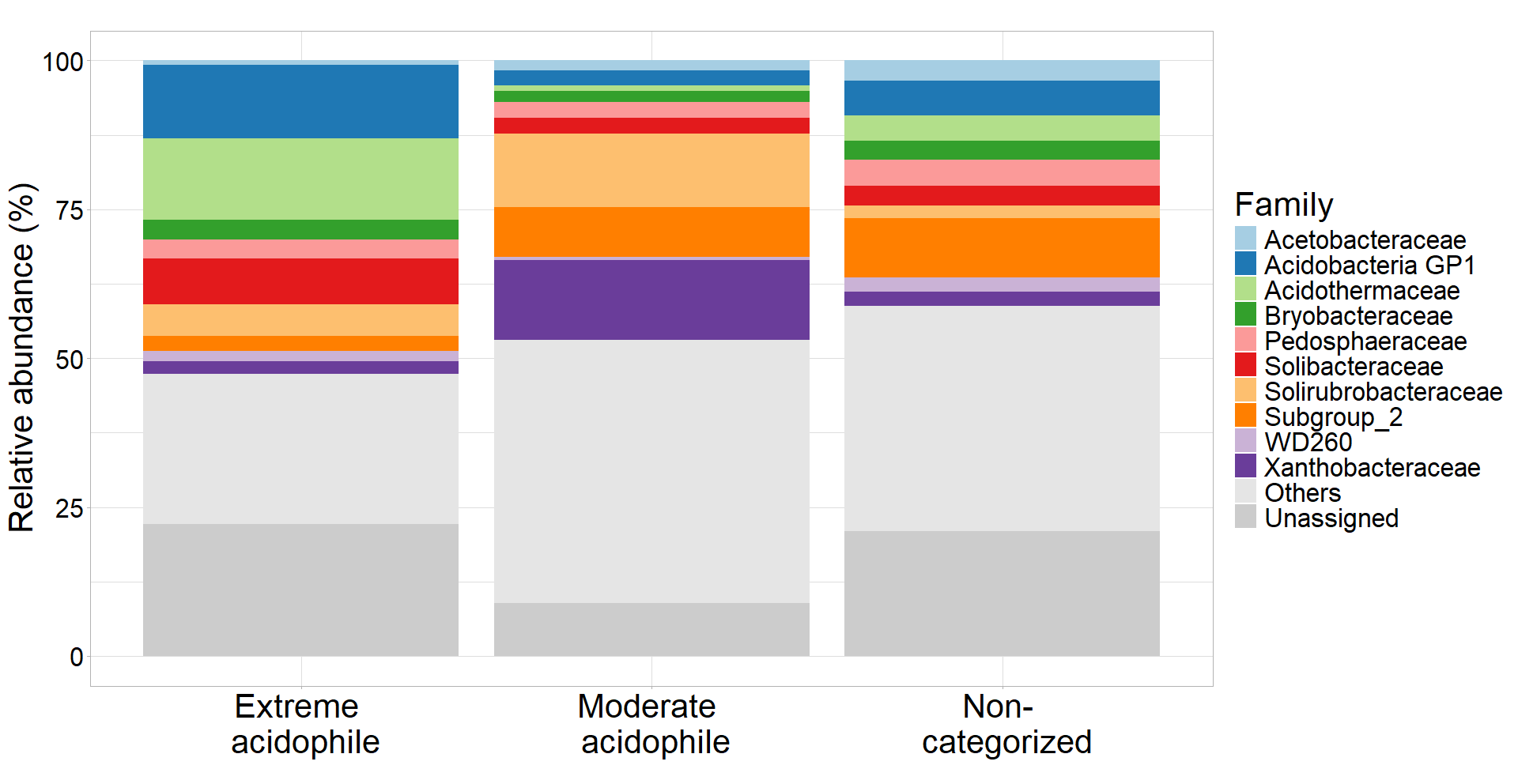


**Figure S2.** Mean relative abundance of the top 10 bacterial families for each LDA category.
