# Supplementary material for: Further reduction in soil bacterial diversity under severe acidification in European temperate forests
Source: Eur J Soil Sci. 2024 Nov 8;75(6):e70005. doi: 10.1111/ejss.70005 (PMC11579971; doi:10.1111/ejss.70005)
Supplement: Supplementary file 2 — Supplementary Material 2. [file EJSS-75-e70005-s001.docx]

# **Supplementary material 2**

**Bioinformatics results**

A total number of 12,175,748 reads for the Veluwe NP samples and 25,685,313 reads for the Bavarian NP samples were respectively retained after the bioinformatic analysis of the sequencing data. Wilcoxon rank sum tests showed no statistical differences between the average number of reads between deciduous and coniferous samples in both Veluwe NP (W = 467, *p* > 0.05) and Bavarian NP (W = 918*,* *p* > 0.05). However, the average number of reads per study site did differ significantly (Wilcoxon rank sum test: W = 1112, p-value < 0.001). Rarefaction resulted in a total number of 6,498,147 reads and 11,191,389 reads, respectively, for Veluwe NP and Bavarian NP. We retrieved a total of 13,659 ASVs in Veluwe NP and 36,063 ASVs in Bavarian NP, clustered in 40 phyla and 435 families.

**Linear discriminant analysis results**

The linear discriminant analysis categorised 2,486 ASVs as “extreme acidophile” (ASVs more abundant at the lowest extreme of the gradient, 24.9% of the total reads), 2,712 ASVs as “moderate acidophile” (ASVs more abundant at the highest extreme of the gradient, 12.3% of the total reads), and the remaining 37,749 ASVs as “non-categorized” (ASVs for which the abundance was independent of soil pH, 62.7% of the total reads). The extreme acidophile community was dominated by the families Acidothermaceae (13.58 ± 3.87%; 4.62 – 29.01%), Acidobacteria GP1 (12.37 ± 5.43%; 0.00 – 25.46%) and Solibacteraceae (7.71 ± 4.34%; 0.31 – 35.17%). The predominant families for the moderate acidophile community were Xanthobacteraceae (13.36 ± 8.30%; 2.51 – 40.69%), Solirubrobacteraceae (12.37 ± 15.29%; 0.64 – 72.70%) and Subgroup_2 (8.44 ± 7.35%; 0.00 – 28.59%). Finally, the top 3 families in the non-categorized community were Subgroup_2 (10.05 ± 4.75%; 2.39 – 35.01%), Acidobacteria GP1 (5.94 ± 5.11%; 0.54 – 24.98%) and Pedosphaeraceae (4.37 ± 2.04%; 0.84 – 13.02%).
